# Proteomic Analysis of Human Follicular Fluid-Derived Exosomes Reveals That Insufficient Folliculogenesis in Aging Women is Associated With Infertility

**DOI:** 10.1016/j.mcpro.2025.100930

**Published:** 2025-02-28

**Authors:** Zhen Liu, Qilin Zhou, Jun Zan, Jingyan Tian, Yangzhuohan Zhang, Fanggui Wu, Huan Zhao, Qianwen Peng, Shangjie Liu, Qianjun Chen, Endong Liu, Zhengdong Liao, Pengfei Zou, Lin Mei, Wen Wang, Sen Dong, Luo Niu, Shengda Wu, Liangge He, Xiaoyi Zhou, Yanbo Jin, Panpan Li, Sheng Yang

**Affiliations:** 1 The Reproductive Medicine Center, The Third Affiliated Hospital of Shenzhen University, Shenzhen Luohu Hospital Group, Shenzhen, China; 2 Guangdong Key Laboratory for Biomedical Measurements and Ultrasound Imaging, National-Regional Key Technology Engineering Laboratory for Medical Ultrasound, School of Biomedical Engineering, Shenzhen University Medical School, Shenzhen, China; 3 Institute of Additive Manufacturing, Jiangxi University of Science and Technology, Nanchang, China; 4 Department of Clinical Medicine, Hubei University of Science and Technology, Xianning, China; 5 Shenzhen University Medical School, Shenzhen University General Hospital, Shenzhen, China

**Keywords:** aging, exosomes, folliculogenesis, in vitro fertilization, proteomic

## Abstract

Although the risk of female infertility increases with advancing age, the underlying mechanisms remain unknown. Exosomes in follicular fluid are suggested to regulate folliculogenesis and influence oocyte quality, potentially playing a critical role in age-related infertility. Elucidating their content could enhance the understanding of the molecular mechanisms associated with female aging-induced infertility. In this study, we explored the proteomic profiles of exosomes derived from human follicular fluid to identify protein signatures associated with infertility in both young and aging women. Despite the lack of significant differences in the morphology and particle size of follicular fluid-derived exosomes between the two groups, proteomic analysis revealed a distinct pattern of differentially expressed proteins (DEPs). DEPs associated with B-cell activation, pathogen invasion, and disrupted metabolic processes were significantly more highly expressed in the aging group than in the young group, indicating their involvement in age-related infertility. *In vivo* experiments demonstrated that the application of exosomes, particularly those derived from young female group, facilitated the successful maturation of follicles. Key exosomal proteins, including ENO1, HSP90B1, fetuin-B, C7, and APOC4, were found to be associated with follicular maturation. Furthermore, the PI3K/AKT signaling pathway, which is known to be related to folliculogenesis, was activated by the application of exosomes in aging female mice. This study provides novel insights into the aging-associated protein signatures of follicular fluid-derived exosomes and their potential role in infertility. These findings suggest that aging-related protein signatures in exosomes could contribute to the treatment of age-related infertility.

## Highlights

• The proteomic landscape of follicular fluid-derived exosomes from young and aging women was determined.
• Increased inflammation and dysfunctional metabolism were found in the follicular microenvironment of aging women.
• Discovered key proteins associated with successful folliculogenesis after ovulation.
• Follicular fluid-derived exosomes regulate folliculogenesis through the PI3K/AKT signaling pathway.

## Graphical Abstract

**Figure:**
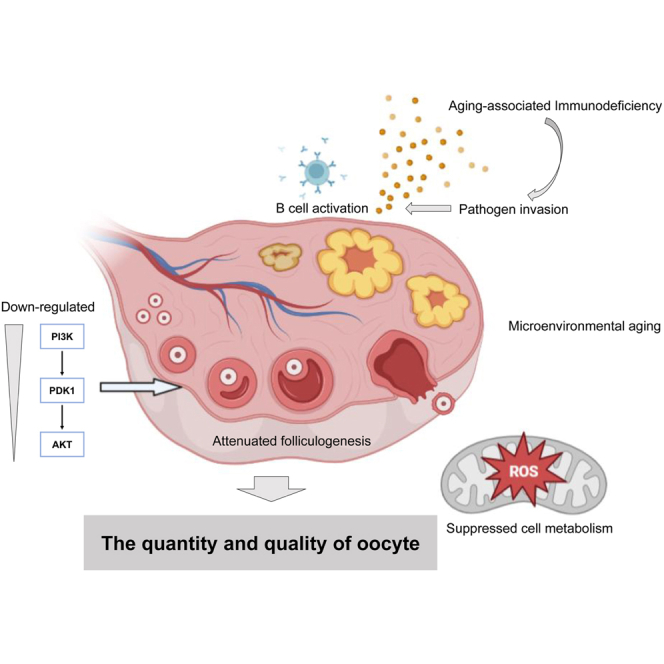

## In brief

In this study, the proteomic profiles of exosomes derived from the follicular fluid of young and aging women were characterized. The aging group exhibited increased inflammation and decreased metabolism, as revealed by our proteomic analysis. Six proteins were identified to be associated with favorable clinical IVF outcomes, with ENO1, HSP90B1, and FETUB suggested to be linked to successful folliculogenesis following ovulation. Subsequent investigations confirmed the attenuation of the PI3K/AKT signaling pathway in the follicles of aging women, which was regulated by follicular fluid-derived exosomes. These findings provide valuable insights for further analysis of the molecular mechanisms by which exosomal proteins affect oocyte quality and support the potential future use of exosomes as a treatment method.

Infertility is a reproductive disorder characterized by the inability to establish a clinical pregnancy through 12 months of unprotected sexual intercourse (1). The World Health Organization (WHO) reported that infertility affects between 8% and 12% of couples worldwide, with rates as high as 30% in some regions (2, 3).

Aging has been identified as the primary factor affecting female fertility; the quality and quantity of oocytes decrease as women grow older (4). Oocyte maturation occurs in the follicle, surrounded by squamous granulosa cells, and the follicular fluid, which represents the microenvironment during oocyte development, is believed to play a critical role in determining the quality of the oocyte (5, 6). While previous studies have identified the protein profile in follicular fluid (7), more recent proteomic analyses have focused on identifying the signaling pathways that contribute to the successful maturation of oocytes (8, 9, 10). A recent study also applied a proteomic strategy to elucidate the mechanism underlying disease-induced infertility, but changes in the protein signature of follicular fluid with aging have not been reported (11, 12). The composition of fatty acids, hormones, and other proteins in follicular fluid strongly contributes to follicle development and oocyte maturation (13). However, the analysis of whole proteins in follicular fluid may be limited by the presence of cellular debris, including apoptotic material and metabolic waste products. These products can prevent the identification and quantification of the critical proteins that participate in communication among oocytes, cumulus cells, and granulosa cells.

Exosomes are spherical lipid bilayer nanoparticles with diameters ranging from 40 to 100 nm (14). These nanoparticles carry proteins, including transmembrane proteins such as CD63 and CD81, and are involved in intercellular communication and material exchange (15). The presence of exosomes in bovine follicular fluid was first identified in 2012 by Gerrit J. Bouma's group, and soon after, exosomes were confirmed to exist in human follicular fluid and were suggested to be involved in follicular maturation (16, 17). Research has shown that exosomes in follicular fluid are involved in communication between oocytes and the surrounding cumulus cells (18, 19, 20). The miRNA profile of exosomes derived from follicular fluid has been characterized in previous studies, and the mechanistic role of this profile has been investigated in disease models such as polycystic ovary syndrome (PCOS) models (17, 21, 22). However, our understanding of the proteomic profile and the underlying mechanisms of the impacts of exosomal proteins on ovary function and oocyte quality remains limited and is primarily based on studies utilizing animal models (23, 24). The current knowledge gap highlights the need for further research into the proteomic profile of human follicular fluid-derived exosomes. By further elucidating the proteomic landscape and functional mechanisms of these exosomes, we may be able to identify new targets for the diagnosis and treatment of reproductive disorders.

In the present study, we elucidated the proteomic profile of human follicular fluid-derived exosomes. Based on our sample population dataset, we identified aging-associated proteins and explored the underlying molecular mechanisms.

## Experimental Procedures

### Ethical Statements

For the human study, we obtained written informed consent and received approval from the Research Ethics Committee of the Third Affiliated Hospital of Shenzhen University, with authorization protocol number 2021-LHRMYY-SZLL-010 and abide by the Declaration of Helsinki principles. All animal protocols received approval from the Committee on the Ethics of Animal Experiments at Zhongxun Precision Medicine Research Institute with statement number A202301083.

### Human Participants

In this study, volunteers were recruited from patients who underwent IVF at the Reproductive Medicine Center of the Third Affiliated Hospital of Shenzhen University. This study was authorized by the Institutional Review Board and Ethics Committee of the Third Affiliated Hospital of Shenzhen University. The patients enrolled in this study provided informed consent. Volunteers suffering from gynecological diseases were excluded. The criteria for the young group were age <28 years and anti-mullerian hormone (AMH) > 2 ng/ml, whereas those for the aging group were age >40 years and AMH <0.5 ng/ml. Follicular fluid was collected by transvaginal ultrasound-guided aspiration 34 to 36 h after human chorionic gonadotropin administration. Only clear fluid without blood or flushing medium contamination was collected. After oocyte isolation, the follicular fluid samples were centrifuged at 1000*g* for 10 min at 4 °C to remove the cellular components and debris. The supernatants were stored at −80 °C before further processing. Seven samples from each group were collected.

### Cell Culture

The human ovarian granulosa cell line KGN and primary granulosa cells were generously provided by Qilin Zhou from the Reproductive Medicine Center at the Third Affiliated Hospital of Shenzhen University. These cells were cultured in 6 cm dishes (Biofil) with DMEM/F12 medium (Gibco) supplemented with 10% fetal bovine serum (Procell) and maintained in an incubator at 37 °C with a 5% CO_2_ atmosphere. The culture medium was changed every 2 days. After reaching 90% confluence, the cells were dissociated with 0.25% trypsin–EDTA (SH30042.02, HyClone) and seeded into 6-well plates or 96-well plates for subsequent experiments or passaged at a 1:3 ratio.

### Isolation of Exosomes From Follicular Fluid

The same standard inclusion and exclusion criteria used for the humans were applied to the participants. A total of 15 to 20 ml of follicular fluid was collected from both the young and the aging groups. The collected follicular fluid was transfered to a 100 kDa ultrafiltration tube (Merck, USA) for centrifugation (5000*g*, 30 min) to condense the fluid. The concentrated fluid was further placed in a 15-ml centrifuge tube and centrifuged at 3000*g* for 15 min to remove residual cells and cell debris. Then, the supernatant was transferred to a fresh 15-ml centrifuge tube and centrifuged at 10,000*g* for 15 min to remove apoptotic bodies, RNA, and proteins. Finally, the supernatant was transferred to an ultracentrifuge tube and centrifuged at 100,000*g* for 2 h. The supernatant was aspirated, and the exosome pellets were resuspended in 100 to 500 μl of PBS. The extracted exosomes were stored frozen at −80 °C for further analysis.

### Nanoparticle Tracking Analysis (NTA)

In accordance with standard procedures, standard samples with a particle concentration of 2.01 × 10^10^ particles/ml, featuring sizes of 68, 91, 113, and 155 nm, were assessed prior to the introduction of the tested exosomes. The exosomes suspended in PBS (SH30256.01, HyClone, USA) were subsequently incrementally diluted with PBS to achieve a particle concentration of 2.01 × 10^10^ particles/ml. These exosome samples were then subjected to analysis using nanoparticle tracking flow cytometry with a NanoFCM N30E instrument (NanoFCM, UK). The size distribution and concentration were obtained from the instrument and further analyzed using Origin 2018 (OriginLab Corporation, USA) and GraphPad Prism 8 (GraphPad Software).

### Transmission Electron Microscopy (TEM)

The shape and size of the exosomes were examined using TEM (HT7700, Hitachi). The PBS-suspended exosomes (10 μl) were loaded on copper mesh and precipitated for 1 min, after which the floating liquid was removed via filter paper. Phosphotungstic acid (10 μl) was loaded on the copper mesh and precipitated for 1 min, followed by removal of the floating liquid using filter paper. The mixture was dried at room temperature for 5 min and imaged at 100 kV.

### Uptake of PKH26-Stained Follicular Fluid-Derived Exosomes

Primary granulosa cells were cultured *in vitro* in DMEM/F12 supplemented with 10% fetal bovine serum and 1% penicillin and streptomycin. Exosomes obtained by ultracentrifugation were labeled with the red fluorescent dye PKH26 (MINI26, Sigma, USA) at room temperature and blocked with an FBS: PBS (v/v, 10%/90%) solution. The solution containing the labeled exosomes was added to a size exclusion column (IZON, New Zealand) to remove the nonconjugated dye and purify the labeled exosomes. The labeled exosomes were added to the culture medium and incubated with granulosa cells. The cells were fixed with 4% formaldehyde at different time points to detect the uptake of the exosomes over time. Nuclear staining was conducted using Hoechst 33342 (23491–45–4; Merck). Exosomes entering cells were observed with a laser scanning confocal microscope (Zeiss, LSM800).

### Label-Free Quantitative Proteomics

Trypsin was used to generate peptides, with up to two missed cleavages permitted. The protein solution (200 μl) was mixed with four volumes of cold acetone containing 30 mM DTT, and the mixture was precipitated at −20 °C for 2 h. After centrifugation at 13,000*g* for 10 min at 4 °C, the supernatant was discarded. The precipitate was resuspended in cold acetone with 10 mM DTT, thoroughly disrupted, vortexed, and incubated at −20 °C for another 2 h. Following another centrifugation step under the same conditions, the supernatant was discarded, and the remaining precipitate was air-dried. U2 lysis buffer (8 M urea, 10 mM EDTA) was added, and the sample was sonicated for 5 min. After centrifugation at 13,000*g* and 4 °C, the supernatant was removed.

Subsequently, 100 μg of protein was dissolved in U2 lysis buffer at a concentration of ∼1 μg/μl. Five volumes of 100 mM TEAB were added, resulting in a 6-fold dilution of the protein. A total of 1.2 μl of 0.5 M CaCl2 was added to the mixture, followed by gentle shaking and centrifugation. Trypsin was added at a trypsin-to-protein ratio of 1:100 for digestion, and the mixture was incubated at 37 °C for at least 8 h. For each 100 μg of peptide, 10 mg of C18 column material was used. The column material was activated with 1 ml of methanol and centrifuged, and the supernatant was discarded. Next, 1 ml of 0.1% formic acid (FA) was added to acidify the sample, which was followed by centrifugation and removal of the supernatant. The peptide samples were further acidified by adding an equal volume of 0.1% FA, vortexed, and gently mixed for 30 min. After centrifugation, the supernatant was discarded, and the samples were washed twice with 0.1% FA + 3% acetonitrile (ACN) to desalt them. The peptides were then eluted with 1 ml of 0.1% FA + 80% ACN and dried via a vacuum concentrator.

### LC/MS Analysis

Peptide samples were diluted to 1 μg/μl in buffer, the sample volume was set to 5 μl, and the scanning mode was 120 min. Peptides with a mass‒charge ratio of 350-1500 in the sample were scanned. Mobile phase A solutions (98% water, 2% ACN, 0.1% FA) and mobile phase B solutions (98% ACN, 2% water, 0.1% FA) were prepared. A precolumn (300 μm × 0.5 mm, 3 μm) and analytical column (3 μm, 75 μm × 150 mm; Welch Materials, Inc.) were used. The spray voltage was 1.9 kV, and peptides separated by liquid-phase chromatography were ionized by a nanoESI source before entry into the Q Exactive HFX tandem mass spectrometer (Thermo Fisher Scientific, San Jose, A) for detection, with the following main parameter settings: ion transfer tube temperature, 320 °C; scanning range, 350–1500 m/z; primary resolution, 60,000; C-Trap3e6, IT 80 ms; secondary resolution, 15,000; C-Trap1e5, IT 100 ms, CE28; threshold intensity, 104; and dynamic exclusion, 30 s. The UniProt human proteome database (HT20220825105011.fasta), containing 79,283 entries, was used. The mass tolerance for precursor ions was set at 20 ppm for the first search and 4.5 ppm for the main search. The mass tolerance for fragment ions was set at 0.5 Da. Carbamidomethylation of cysteine was set as a fixed modification, whereas oxidation of methionine and N-terminal acetylation were set as variable modifications. The FDR was set at 1% at both the peptide and protein levels. Label-free quantification (LFQ) was employed using the MaxLFQ algorithm integrated into MaxQuant. Raw data for mass detection (raw) were generated. MaxQuant 1.6.15.0 was used to retrieve and analyze the mass spectrometry data. The criteria for significant differences were upregulation ≥1.2 or downregulation ≤0.833, with an adjusted *p* value ≤ 0.05. The identified proteins were listed in supplemental Table S1.

### Gene Ontology (GO) and Kyoto Encyclopedia of Genes and Genomes (KEGG) Pathway Enrichment Analyses

GO and KEGG pathway enrichment analyses of the target proteins were performed with DAVID (https://david.ncifcrf.gov/; release-2021_04/). The statistical test used here is based on the equation *p* = 1 − ∑ i = 0 m − 1 (M i) (N − M n − i) (N i). N represents the number of proteins with GO annotation information among all proteins, n represents the number of differentially expressed proteins (DEPs) in N, M represents the number of proteins annotated to a specific GO entry among all proteins, and m represents the number of DEPs annotated to a specific GO entry. The screening criterion was *p* value < 0.05.

### Experimental Animals

The mice were obtained from Vital River Laboratories and housed in the animal facility at Shenzhen Zhongxun Precision Medicine Research Institute. The mice were maintained under a 12 h light/12 h dark cycle at 22 °C with free access to food and water. The ovaries of C57BL/6 female (8 months old) mice treated with or without exosomes were collected as described for the aging group (n = 13). C57BL/6 female mice (2 months) were used as the young control group (n = 3). Ovaries of 10-day-old (P10) C57BL/6 female mice were used for *in vitro* folliculogenesis (n = 24).

### Treatment With Follicular Fluid-Derived Exosomes (FF-exos)

The FF-exos, which were isolated from either young or aging women, were diluted to a concentration of 10 μg/ml (corresponding to 1 × 10ˆ10 particles/ml) in PBS. Subsequently, 100 μl of the diluted exosomes was administered to C57BL/6 female mice via tail vein injection once a week for 2 weeks. The mice were anesthetized before being sacrificed, and their ovaries were collected postmortem for subsequent hematoxylin and eosin (HE) staining and immunohistochemical analysis.

### *In vitro* Folliculogenesis

P10 ovaries were harvested and cultured in 24-well plates (TCP000024, Biofil) with 0.3 ml of culture medium added to the bottom of each well. The culture medium consisted of minimum essential medium (MEM)-alpha (C11095500BT, Gibco) supplemented with 0.23 mM pyruvic acid (V900232–100G, Sigma), 50 mg/L streptomycin sulfate (Gibco, USA), 75 mg/L penicillin G (Gibco), and 3 mg/ml BSA (164,210, Procell). The ovaries were randomly allocated to the control and treatment groups, with each group containing two to three ovaries. Ovaries cocultured with 0.03 U/ml follicle-stimulating hormone (FSH) (GP21245–100; GlpBio) served as controls. The ovaries in the YFF group and AFF group were treated with 10 μg/ml follicular fluid-derived exosomes from young women (YFF-exos) and follicular fluid-derived exosomes from aging women (AFF-exos), respectively, while they were cocultured with FSH. The ovaries were cultured for 96 h, and the medium and exosomes were changed every 2 days. The ovaries were further collected for HE staining to evaluate follicle development.

### HE Staining

For HE (C0105M, Beyotime) staining, the 15th ovary slice were used from each group. For hematoxylin staining, the tissues were exposed to the stain at room temperature for approximately 5 to 10 min, enabling visualization of the cell nuclei. For eosin staining, the tissues were subsequently subjected to staining at room temperature for 1 to 3 min, resulting in the coloration of the cytoplasmic components and the extracellular matrix.

### Immunohistochemistry

After natural cooling, the slides were placed in PBS (pH 7.4) and agitated on a decolorizing shaker for three washes, each lasting 5 min. The slides were submerged in a 3% hydrogen peroxide solution and incubated at room temperature in the dark for 25 min. Then, the slides were returned to PBS (pH 7.4) and subjected to three washes on a decolorizing shaker, each lasting 5 min. Then, 3% BSA (B2064–1, Sigma, USA) was applied evenly to cover the tissue, and the tissue was blocked at room temperature for 30 min. Primary antibodies were applied at a 1:100 ratio with PBS to the slides, which were placed flat in a humid chamber and incubated overnight at 4 °C. The primary antibodies used were as follows: anti-PI3K (20584-1-AP; Proteintech), anti-AKT (60203-2-Ig; Proteintech), anti-pAKT (66444-1-Ig; Proteintech, USA), anti-ENO1 (1:1,000, 11204-1-AP, Proteintech), anti-FETUB (1:1,000, 67002-1-Ig, Proteintech, USA), anti-APOC4 (1:1,000, 16530-1-AP, Proteintech, USA), anti-HSP90B1 (1:1,000, 114052-1-AP, Proteintech), and anti-C7-500, 17642-1-AP, Proteintech). The slides were subsequently placed in PBS (pH 7.4) and agitated on a decolorizing shaker for three washes, each lasting 5 min. After the excess liquid was gently removed, the appropriate secondary antibody (HRP-labeled), which corresponded to the primary antibody species, was added, and the sections were incubated at room temperature for 50 min. Another set of three 5 min washes was performed on the slides with PBS (pH 7.4), and the excess liquid was gently shaken off. Next, freshly prepared DAB (D12384, Sigma) staining solution was applied. The staining duration was controlled under a microscope; positive results manifested as a brownish-yellow color. The staining process was terminated by rinsing the slides with tap water.

### Follicle Counting

*In vitro* cultured ovaries and ovaries from each experimental group were collected and fixed in 10% buffered formalin overnight for serial sectioning (5 μm) and HE staining. All follicles were enumerated at every fifth section, guided by their phenotypic characteristics as previously reported in the literature (25, 26). Two independent observers performed the section counts for comparative analysis.

### Cell Counting Kit-8 (CCK-8) Cell Proliferation Assay

In brief, human primary granulosa cells and KGN granulosa cells were cultured in 96-well plates at a density of 5 × 10^3^ cells per well in 100 μl of cell culture medium. Following treatment with exosomes, 10 μl of CCK-8 solution (GK10001, GlpBio) was added to each well. The cells were then incubated at 37 °C for 2 h, and the optical density was subsequently measured at a wavelength of 450 nm using a microplate reader.

### Western Blotting

RIPA lysis buffer (Beyotime) was added to the exosome suspension (v/v, 33%/66%), and the mixture was placed on ice for lysis for 30 min. The protein concentration was measured via the BCA (23,227, Thermo) method. Each sample contained an equal amount of protein, which was mixed with loading buffer and boiled for 10 min to denature the protein for protein electrophoresis. When the ladder showed protein samples entering the separation gel, the voltage was increased to 120 V. Electrophoresis was stopped when the bromophenol blue strip reached the bottom of the glass plate. The proteins were transferred to a PVDF membrane at 30 mA for 90 min. The blot was blocked with 5% BSA blocking solution on a shaker at room temperature for 2 h. The following primary antibodies were used: anti-TSG101 (1:500, 28283-1-AP, Proteintech), anti-CD81 (1:500, 66,866-1-lg, Proteintech), anti-CD63 (1:500, 25682-1-AP, Proteintech), anti-CD9 (1:250, 20597-1-AP, Proteintech), anti-ENO1 (1:1,000, 11204-1-AP, Proteintech), anti-FETUB (1:1,000, 67002-1-Ig, Proteintech), anti-APOC4 (1:1,000, 16530-1-AP, Proteintech), anti-HSP90B1 (1:1,000, 114052-1-AP, Proteintech), anti-C7-500, 17642-1-AP, Proteintech), anti-GAPDH (1:1,000, 60004-1-Ig, Proteintech), and anti-Alix (1:1000, 12422-1-AP, Proteintech). The blot was incubated overnight with the primary antibodies in a shaker at 4 °C. The next day, the membrane was washed 3 times with Tris-buffered saline (TBS) supplemented with 0.1% TBST on a shaker for 10 min each. The corresponding secondary antibodies (HRP-conjugated goat anti-mouse and HRP-conjugated goat anti-rabbit antibodies, Proteintech) were applied at a dilution of 1:10,000, and the membrane was incubated at room temperature on a shaker for 1 h. The secondary antibody solution was then aspirated, and TBST was added to wash the PVDF membrane 3 times for 10 min each. Chemiluminescence was detected using Pierce ECL (36208ES60, Yeasen) combined with Western blotting substrate.

### Random Forest Algorithm

The random forest algorithm integrates multiple decision trees through ensemble learning. Each decision tree in the algorithm is constructed using a random vector, with all vectors in the random forest being independently and identically distributed. This method introduces randomness to both the column variables and row observations of the dataset, generating multiple classification categories. The final classification outcome is obtained by aggregating the results from all decision trees. In this study, data from the proteomic cohorts were analyzed using a random forest classifier (27). The expression levels of 69 differentially expressed proteins were used as inputs, with samples from two groups, young and aging women. The model was implemented using the 'RandomForest' package in R, with the default parameters, except for 'n_trees' (set to 500) and 'max_depth' (set to 10). After performing stratified 10-fold cross-validation, the top 10 proteins were selected for neural network training based on their importance values (cutoff = 1).

### Experimental Design and Statistical Rationale

Follicular fluid samples from female subjects were collected according to the procedures outlined in the Human Subjects section. Volunteers were selected based on the criteria for the young and aging groups, with seven samples obtained from each group. The impact of FF-exos on primary granulosa cells was investigated in three independent experiments. The effects of FF-exos on mice were assessed with more than three biological replicates, with the organs collected simultaneously. Newborn mouse ovaries were collected three times for *in vitro* folliculogenesis. Continuous data are presented as the mean ± SEM. Statistical analyses were performed with GraphPad Prism 8.0. The statistical significance of the test results was evaluated by one-way ANOVA and an unpaired *t* test. A *p* value less than 0.05 was considered to indicate statistical significance.

## Results

### Characterization of Young and Aging Women

In this study, follicular fluid was collected from volunteers who underwent IVF at the Reproductive Medicine Center of the Third Affiliated Hospital of Shenzhen University. The criteria for the young group were age <28 years and AMH >2 ng/ml, whereas those for the aged group were age >40 years and AMH <0.5 ng/ml. Comparisons of the hormone levels and IVF outcomes between the young and aging women are shown in Table 1. Compared with the aging group, the young group presented a significantly shorter duration of infertility, confirming the association between increased infertility rates and aging. Furthermore, significantly greater levels of the hormones AMH and estradiol (E2) were observed in the aging group than in the young group, indicating attenuated ovarian function. The slightly elevated levels of FSH were consistent with previous findings. However, no significant differences were observed in the levels of luteinizing hormone (LH), progesterone, or gonadotropin-releasing hormone (GnRH). Importantly, we collected a significantly greater number of healthy, mature eggs from the young group than from the aging group.

**Table 1:** Baseline characteristics and results of IVF treatment parameters of patients who contributed follicular fluid in this study

Characteristics	Young follicular fluid	Aging follicular fluid	*p* Value
Mean (SD) age, years	26.70 ± 1.252	42.00 ± 2.055	0.027
Mean (SD) duration of infertility, years	3.10 ± 1.524	5.60 ± 3.534	0.002
Mean (SD) AMH	3.92 ± 1.205	0.316 ± 0.106	0.001
bE2 (pg/ml)	30.10 ± 9.243	33.30 ± 22.598	0.004
bFSH (IU/L)	7.367 ± 2.560	13.316 ± 3.287	0.207
bLH (IU/L)	5.107 ± 2.384	4.100 ± 1.770	0.72
bP (ng/ml)	0.613 ± 0.462	0.313 ± 0.241	0.322
Total Gn (IU)	2011.95 ± 575.709	1890.00 ± 448.330	0.536
Egg retrieval	15.90 ± 6.402	2.20 ± 1.229	<0.001

bE2, bFSH, and bP refer to the blood concentrations of the hormones E2, FSH, and progesterone. Gn refers to the blood concentrations of the gonadotropin hormone.

### Exosomal Protein Profiles of the Young and Aged Groups

To confirm the presence of exosomes, we performed Western blot analysis to detect exosome-specific protein markers, including CD9, CD63, CD81, and TSG101 (supplemental Fig. S1*A*). Both groups presented similar expression levels of these markers. The morphological characteristics of the exosomes were assessed via transmission electron microscopy (TEM), which revealed the presence of double-membrane vesicles with a characteristic saucer-like shape, which was consistent with previous descriptions of exosomes (supplemental Fig. S1*B*) (27). Additionally, particle size analysis was conducted to determine the average diameter of the exosomes in each group, which measured 80.99 nm and 83.78 nm, respectively, with no significant differences in size between the groups (supplemental Fig. S1*C*).

The exosomes were then labeled with PKH26 and cocultured with primary granulosa cells for 12 h. Following Hoechst 33,342 staining of the nuclei, fluorescence microscopy confirmed the uptake of the exosomes by the granulosa cells, as evidenced by the red fluorescence observed in the cytoplasm and nuclei (supplemental Fig. S1*D*).

**Fig. 1:**
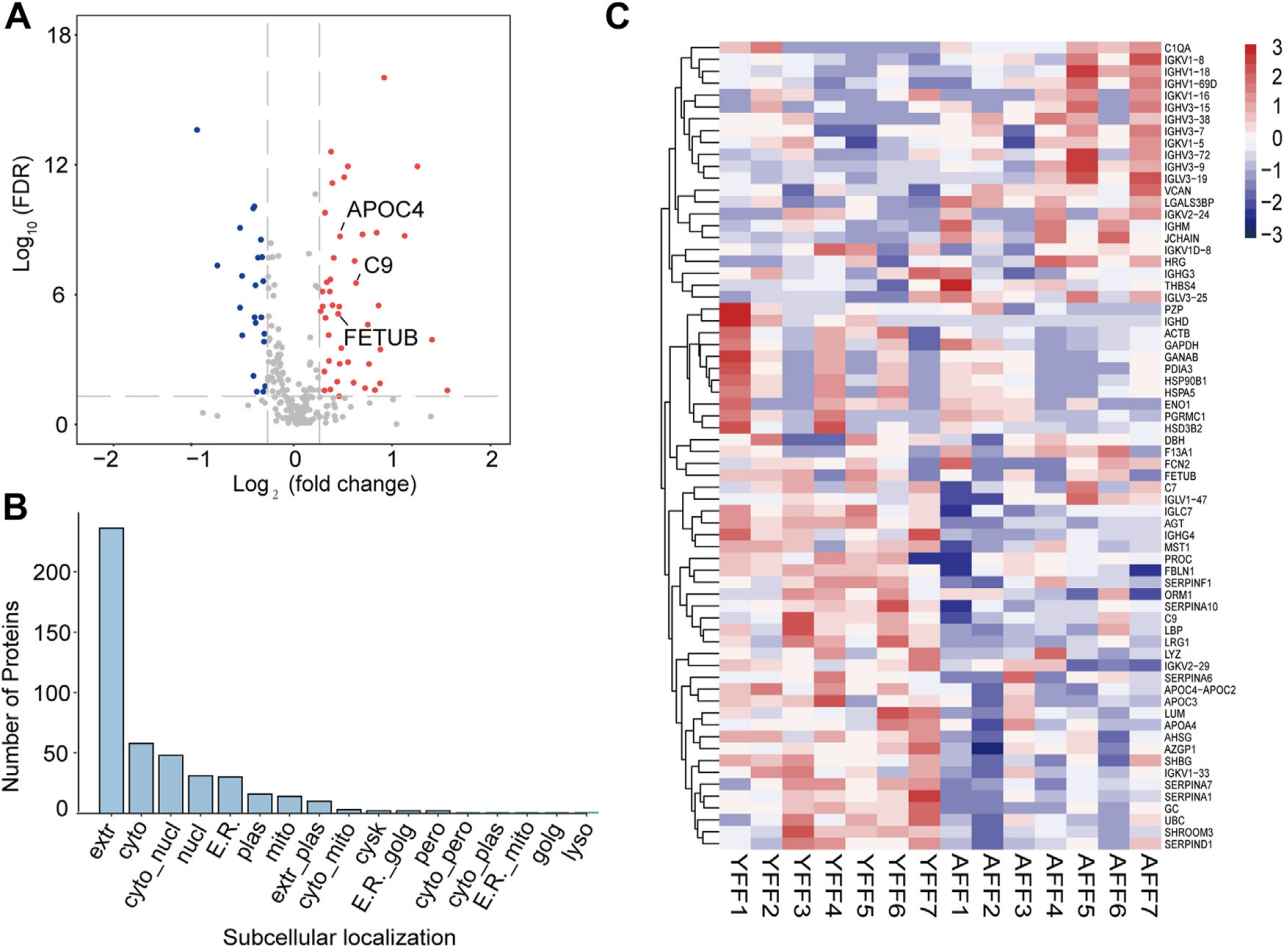
**Proteomic profile of FF-exos from young and aging women.***A*, Volcano plot displaying the DEPs in the two groups (n = 7 each, young vs. aging women), illustrated by plotting the log2-fold change on the x-axis and the -log10-transformed *p* value on the y-axis. Proteins that were upregulated in the young group are shown in *red*, while downregulated proteins are shown in *blue*. The criteria for significant differential expression were a *p* value less than 0.05 and a log2-fold change greater than 0.5. *B*, subcellular localization of the DEPs. *C*, hierarchical clustering analysis of the DEPs in the exosomes of the two groups.

The proteomic profiles of the exosomes from both the young and aging groups were analyzed using label-free quantitative proteomics. Protein abundance was quantified based on normalized areas, and both groups presented comparable protein abundances (supplemental Fig. S2). Principal component analysis (PCA) of exosome-associated proteins separated the samples into two distinct clusters, except for the AFF4 outlier (supplemental Fig. S3). A total of 468 proteins were identified across both groups, of which 69 were differentially expressed (supplemental Table S2). Among these proteins, 46 were upregulated and 23 were downregulated in YFF-derived exosomes (YFF-exos) compared with AFF-derived exosomes (AFF-exos) (Fig. 1*A*). Subcellular localization analysis revealed that most differentially expressed proteins (DEPs) were associated with extracellular vesicles, with a notable proportion localized to the mitochondria or cytoplasm (Fig. 1*B*). Hierarchical clustering further visualized the distinct patterns of upregulated and downregulated proteins between the young and aging groups (Fig. 1*C*).

### DEPs in the Aging Group Were Related to an Activated Immune Response, Suppressed Metabolic Activity, and the PI3K/AKT Signaling Pathway

**Fig. 2:**
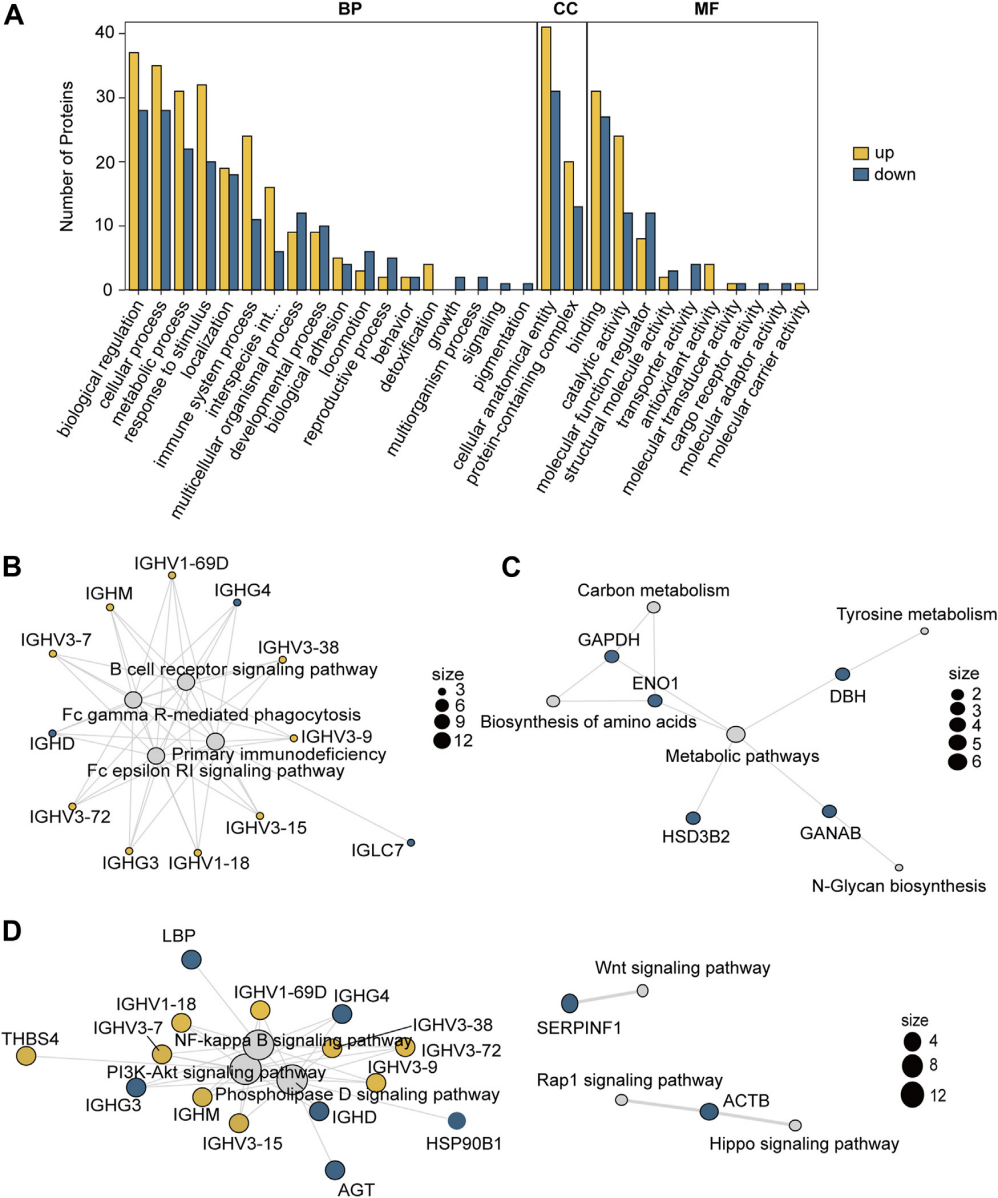
**GO and KEGG analyses of DEPs in FF-exos from young and aging women.***A*, the biological processes associated with the DEPs were analyzed using GO term clustering and are presented in a bar diagram. The upregulated and downregulated bars represent proteins that were either upregulated or downregulated in the two experimental groups. BP refers to a biological process. CC refers to a cellular component. MF refers to molecular function. *B*, the enriched immune-associated signaling pathways associated with the DEPs were identified using KEGG pathway analysis. *C*, the enriched metabolism-associated signaling pathways of the DEPs were identified using KEGG pathway analysis. *D*, the enriched molecular signaling-associated pathways of the DEPs were identified using KEGG pathway analysis.

To identify exosomal biological process changes related to aging, we conducted a GO analysis to identify the specific biological processes associated with the DEPs. The results revealed that the majority of the proteins were involved in immune system processes (21/69) or metabolic processes (18/69) (supplemental Table S3). GO enrichment analysis revealed that the upregulated proteins in exosomes were highly involved in immune response processes, such as the B-cell receptor signaling pathway, positive regulation of B-cell activation, defense response to bacteria, phagocytosis recognition, and blood coagulation (Fig. 2*A*). Furthermore, KEGG analysis revealed that the DEPs were involved in 113 biological processes (supplemental Table S4). Although human disease and organism systems were enriched, the remaining processes were enriched in three main categories: metabolism, the immune system, and signaling pathways. B-cell activation and primary deficiency were the most enriched immune processes and were affected mainly by B-cell VDJ rearrangement fragments such as IGHV and IGHD (Fig. 2*B*). Metabolism involved biological processes such as carbon metabolism, tyrosine metabolism, and amino acid synthesis (Fig. 2*C*). The results revealed that proteins associated with these metabolic processes were expressed at significantly lower levels in the aging group than in the young group. Several signaling pathways, such as the Wnt signaling and Hippo signaling pathways, were suggested to be affected by follicular exosomes, but the PI3K/AKT signaling pathway was found to be most strongly regulated by these DEPs (Fig. 2*D*).

### Exosomes From the aging Group Exhibited Insufficient Folliculogenesis

Recent studies have suggested that PI3K/AKT signaling plays a critical role in folliculogenesis (25). In this study, we aimed to investigate the function of exosomes derived from young and aging individuals in the regulation of folliculogenesis. First, we examined the effects of follicular fluid-derived exosomes (FF-exos) on both primary granulosa cells and KGN granulosa cells *in vitro*. The cells were cocultured with either exosomes from young follicular fluid (YFF-exos) or aging follicular fluid (AFF-exos), with the control group remaining untreated. Proliferation and viability were assessed via CCK-8 assays and MitoTracker staining. The results revealed that YFF-exos did not significantly alter the proliferation or viability of primary or KGN granulosa cells compared with those of the control group (supplemental Fig. S4). However, AFF-exos inhibited the proliferation of KGN granulosa cells, indicating that AFF-exos may suppress cell growth.

**Fig. 3:**
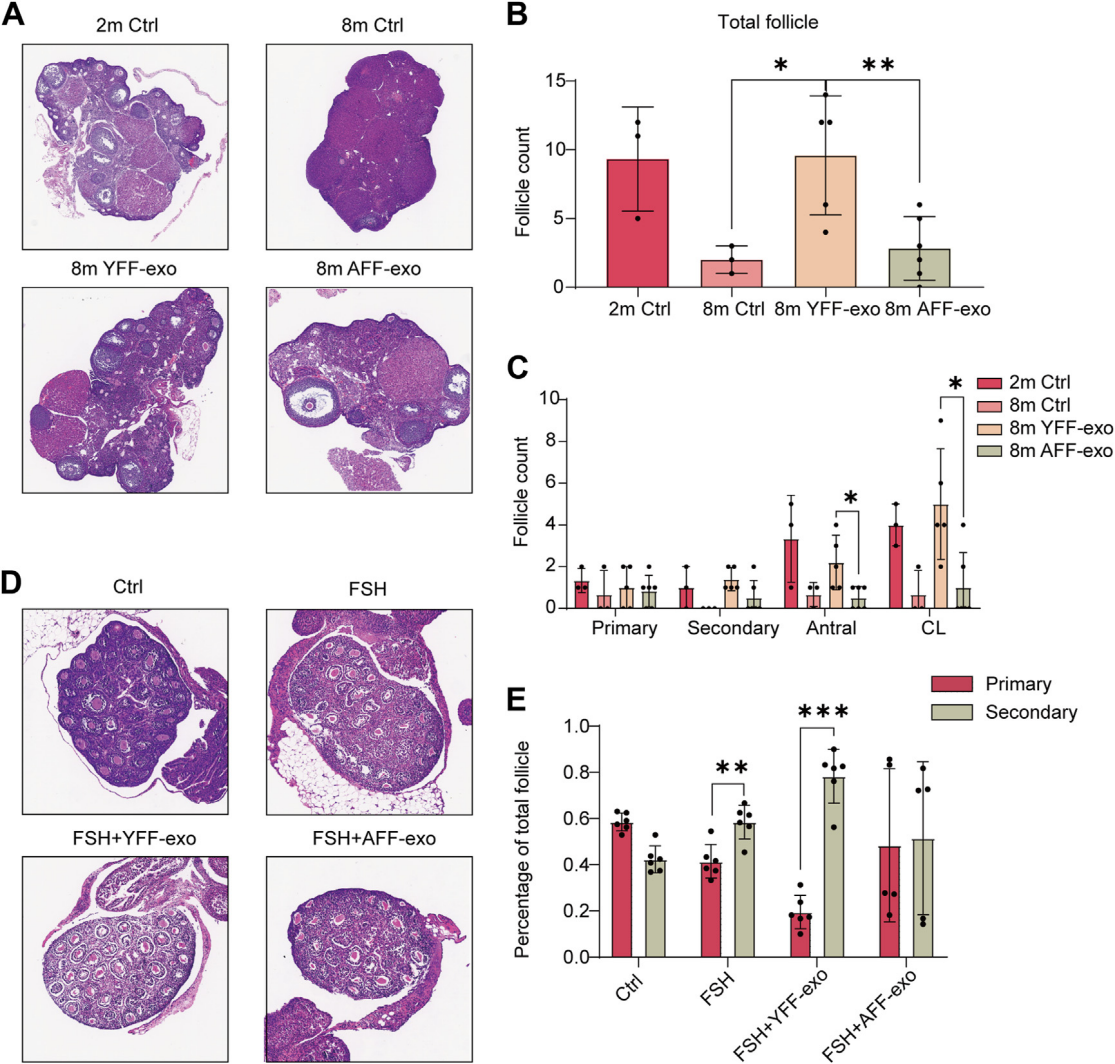
**AFF-exos are incapable of inducing folliculogenesis.***A*, HE staining of ovarian tissue from 2-month-old mice (2 m ctrl), 8-month-old mice (8 m ctrl), 8-month-old mice treated with YFF-exos (8 m YFF-exos), and 8-month-old mice treated with AFF-exos (8 m AFF-exos). *B*, quantification of the total number of follicles across four groups: young control (n = 3), aging control (n = 3), young follicle fluid exosome-treated (YFF-exos) (n = 5), and aged follicle fluid exosome-treated (AFF-exos) (n = 5) groups. The mean value ± standard deviation was plotted. One-way ANOVA with subsequent post hoc analysis using an unpaired *t* test was performed. The calculated *p* value of the YFF-exo group compared with the 8-months control group and the AFF-exo group was 0.0273 and 0.009, respectively. *C*, quantification of follicles at different stages (primary, secondary, antral, and corpus luteum). Young ctrl (n = 3), aged ctrl (n = 3), YFF-exos (n = 5), and AFF-exos (n = 5). The mean value ± standard deviation was plotted. One-way ANOVA with subsequent post hoc analysis using an unpaired *t* test was performed. The *p* values were 0.0169 and 0.0136 from left to right. *D*, HE staining of ovarian tissues from P10 mice. A control group (ctrl) and three treatment groups (FSH, FSH with YFF-exos, and FSH with AFF-exos) were included. *E*, quantification of the percentages of primary and secondary follicles in the four groups (n = 6 for each group). The mean ± SD was plotted. The *p* value of the FSH group was 0.0023. The *p* value of the FSH + YFF-exo group was <0.001.

To establish an *in vivo* aging model, we used 9-month-old female mice. The aging female mice were treated with either YFF-exo or AFF-exo to evaluate the therapeutic effects of exosome application. The ovarian volumes were significantly smaller in the aging group compared to the young group (supplemental Fig. S5*A*). The application of exosomes increased ovarian volume, although the increase was not statistically significant based on the quantification (supplemental Fig. S1*B*). Moreover, the aging group presented a reduced number of ovarian follicles, which is consistent with ovarian dysfunction during aging (Fig. 3*A*). This study aimed to evaluate the therapeutic efficacy of YFF-exos compared with that of AFF-exos. Exosomes were administered via tail vein injections once a week for 2 weeks. Notably, treatment with YFF-exos led to a significant increase in follicle numbers, whereas AFF-exo treatment had no significant effect (Fig. 3*B*). The quantification of follicles at different developmental stages revealed no difference in the number of primary follicles between the groups; however, there were significant differences in the later stages. Specifically, YFF-exo treatment resulted in a marked increase in the number of secondary follicles, which was comparable to that observed in 2-month-old control mice (Fig. 3*C*). In contrast, compared with the 8-month-old control, the AFF-exo treatment did not significantly increase the number of antral follicles and even resulted in a lower count.

To further investigate the role of exosomes in folliculogenesis, we harvested ovaries from postnatal day 10 (P10) mice and assessed the effects of exosomes on follicle development *in vitro*. Ovaries were cultured with FSH to stimulate follicle activation, and exosomes were added simultaneously. After 72 h of coculture, HE staining was performed to evaluate follicle maturation, which progressed from the primary to the secondary stage in all of the experimental groups (Fig. 3*D*). Quantitative analysis revealed a greater percentage of secondary follicles in the YFF-exo group than in the other groups (Fig. 3*E*). In contrast, compared with the FSH control treatment, the AFF-exo treatment impaired folliculogenesis, as indicated by a lower percentage of primary and secondary follicles.

### The Aging-Associated Protein Signature of FF-exos was Related to Unsuccessful IVF

**Fig. 4:**
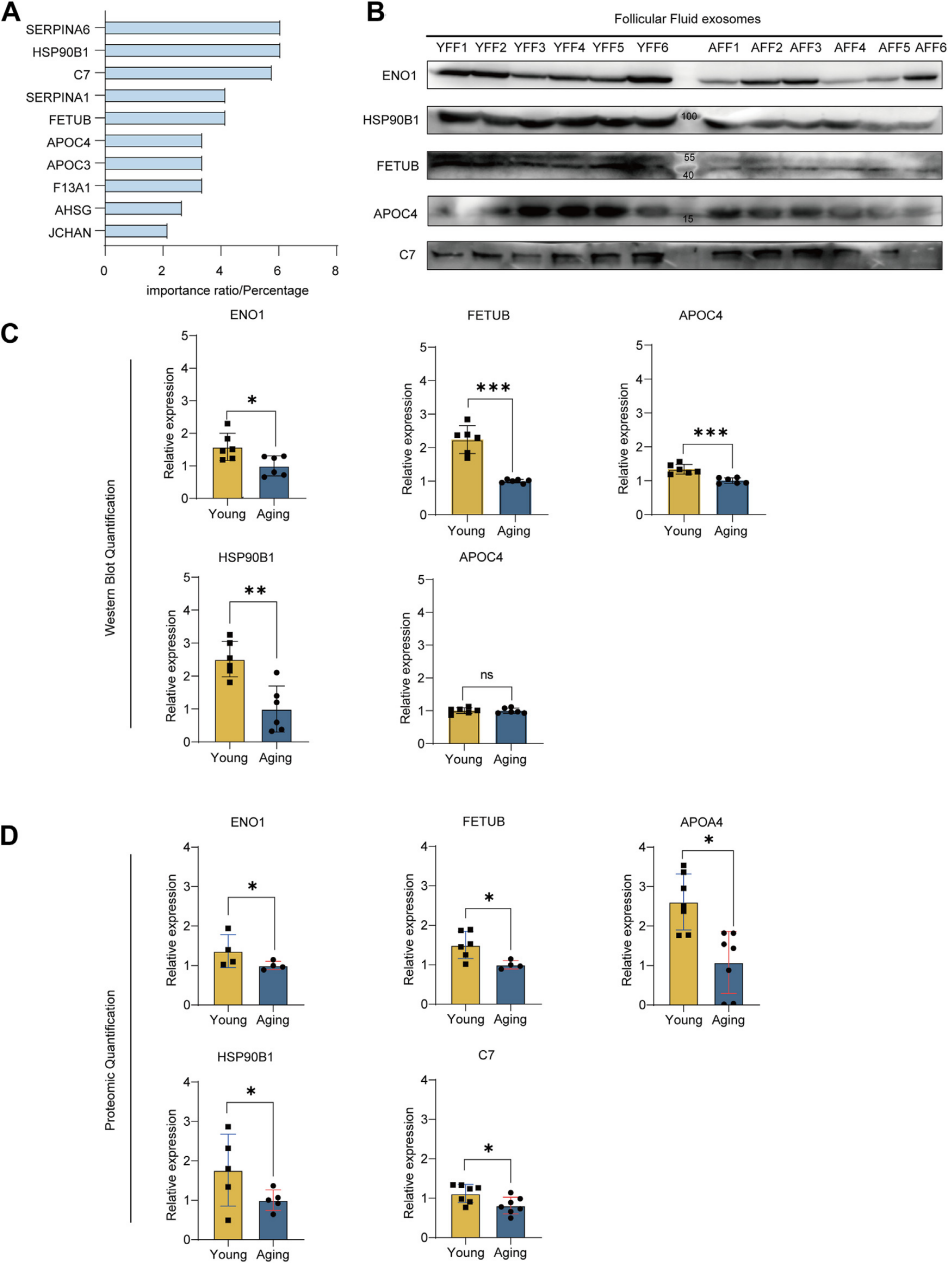
**Aging protein signature of FF-exos associated with successful IVF.***A*, prediction of the top 10 DEPs in FF-exos (n = 7 for both the young and aging groups) using random forest machine learning. *B*, Western blot analysis of key signature proteins, including ENO1, FETUB, APOC4, HSP90B1, and C7. YFF and AFF samples (n = 6 for each group) were collected to isolate the exosomes for immunoblotting. *C*, quantification of blotted protein expression using ImageJ (n = 6). The mean ± standard deviation was plotted. The *p* values for ENO1, FETUB, APOC4, HSP90B1, and C7 were 0.0199, 0.0774, 0.0434, 0.0018, and 0.5186, respectively. *D*, quantification of protein expression in exosomes from the young and aged groups according to the proteomic data (n = 7 for each group). The mean ± standard deviation was plotted. The *p* values of ENO1, FETUB, APOC4, HSP90B1, and C7 were 0.0105, 0.0007, 0.0001, 0.0124, and 0.9209, respectively.

Previous proteomic analyses and *in vivo* experiments have shown that unsuccessful folliculogenesis in aging women is an important factor contributing to infertility. To expand on these findings, we employed a random forest machine learning model to explore the protein signature associated with successful IVF outcomes. Notably, 10 of these proteins were expressed at relatively low levels in AFF-exos and identified to be the important factor affecting IVF outcomes (Fig. 4*A*). We selected the top 10 proteins identified by random forest machine learning that are involved in the molecular signaling pathways identified by previous KEGG analysis. We also included ENO1 is a central protein regulating metabolic processes. Exosomes were isolated from six additional follicular fluid samples from young women and from six additional follicular fluid samples from aging women. Subsequent Western blot analysis revealed differential expression of the key proteins previously identified between the two groups. Specifically, ENO1 and HSP90B1 are downstream signals of PI3K/AKT signaling, whereas APOC4 is involved in lipid metabolism, and C7 functions in the complement signaling pathway. Notably, the protein distribution within each group varied among individuals (Fig. 4*B*). However, further quantitative analysis revealed a deficiency in protein expression, particularly of ENO1, APOC4, and HSP90B1, in the aging group (Fig. 4*C*). We further identified exosomal signature proteins in each sample (supplemental Fig. S6). However, we encountered difficulty identifying a suitable housekeeping protein to demonstrate equal loading of total protein in each sample. Moreover, the quantification of proteomic data indicated a marked reduction in the expression of all proteins in the aging group (Fig. 4*D*).

### Exosomes Deliver the Key Proteins Associated With Folliculogenesis After Ovulation

Previous data verified the differential expression of key proteins between the young and aging groups. To further investigate the functions of these proteins, the delivery of proteins by exosomes to the ovary was tested. Twelve exosome samples were isolated from the young group, and the protein expression levels of ENO1 and HSP90B1 were evaluated by Western blot (supplemental Fig. S7*A*). The exosomes contained varying amounts of each protein, with samples #3, #4, and #12 selected for application in the mouse model. The results indicated that exosomes containing higher amounts of ENO1 compared to the other two groups showed a higher expression of ENO1 in the ovary (supplemental Fig. S7*B*). Quantification of the IHC staining suggested that exosomes could deliver specific proteins to the ovary via tail vein injection, and this delivery was not affected by the quantities of other proteins (supplemental Fig. S7*C*).

**Fig. 5:**
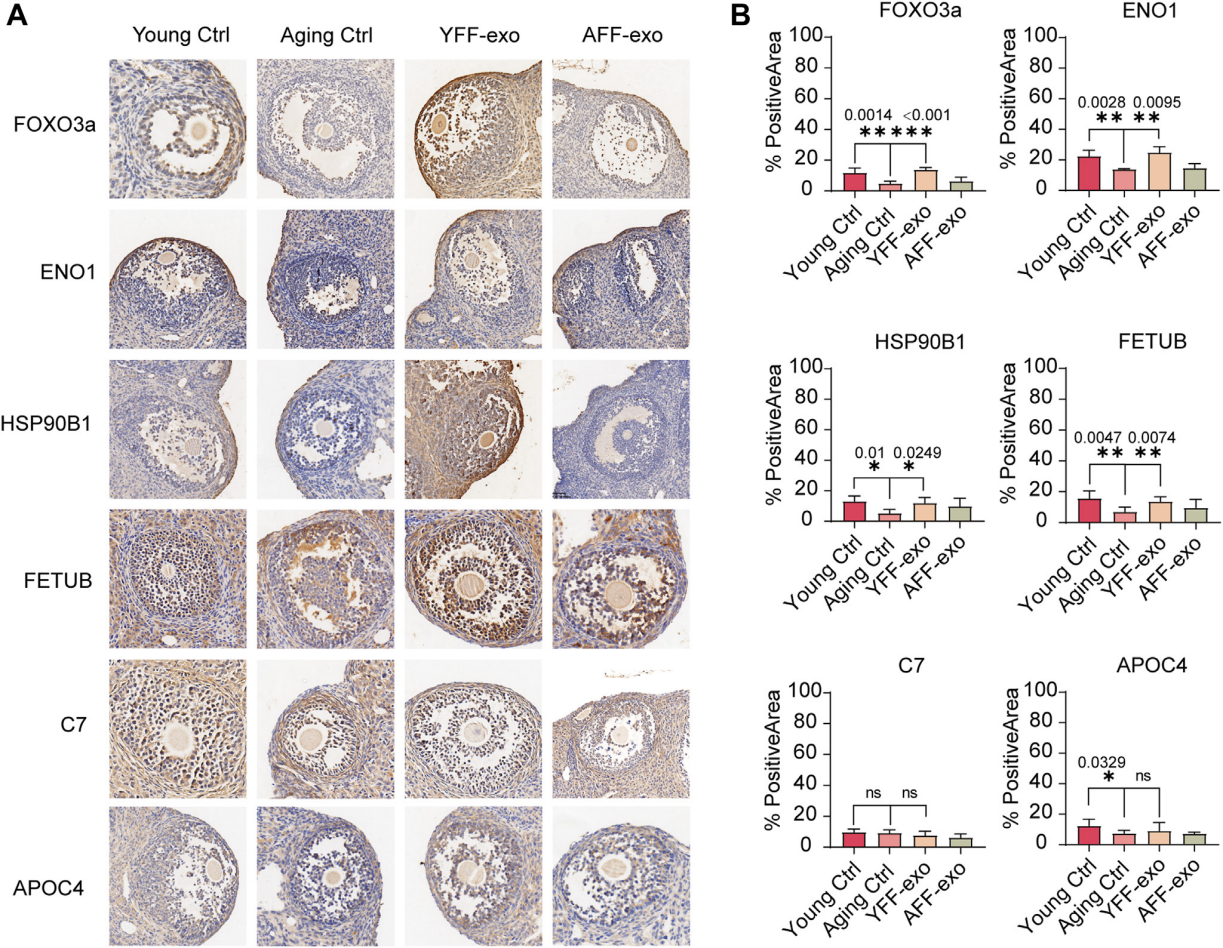
**Exosomal key proteins associated with successful folliculogenesis.***A*, the expression levels of FOXO3a, ENO1, HSP90B1, FETUB, C7, and APOC4 are illustrated in the image showing the IHC results (n = 4 for each group). Bar = 100 μm. *B*, the expression of these proteins in the follicles was quantified using ImageJ software. The *p*-values are annotated above the SD bars.

The expression of these key proteins was further analyzed using IHC staining to compare their expression between groups. The results show that the expression of ENO1, HSP90B1, FETUB, and APOC4 proteins was higher in the antral follicles of the young group compared to the aging group (Fig. 5*A*). However, the expression of C7 did not show a significant difference between groups. It was also found that the expression of ENO1, HSP90B1, and FETUB proteins, except for APOC4, was higher in follicles after exosome treatment compared to the aging group. FOXO3a is a protein that indicates successful follicle maturation. The expression of FOXO3a was significantly higher after exosome treatment, suggesting that the application of exosomes promotes follicle maturation following ovulation. Moreover, quantification of the expression of ENO1, HSP90B1, and FETUB proteins, showing a pattern similar to FOXO3a expression across these groups, suggests that these proteins might participate in promoting follicle maturation through exosome delivery to the ovary (Fig. 5*B*).

### Exosomes Treatment Activates the PI3K/Akt Signaling Pathway to Promote Folliculogenesis After Ovulation

**Fig. 6:**
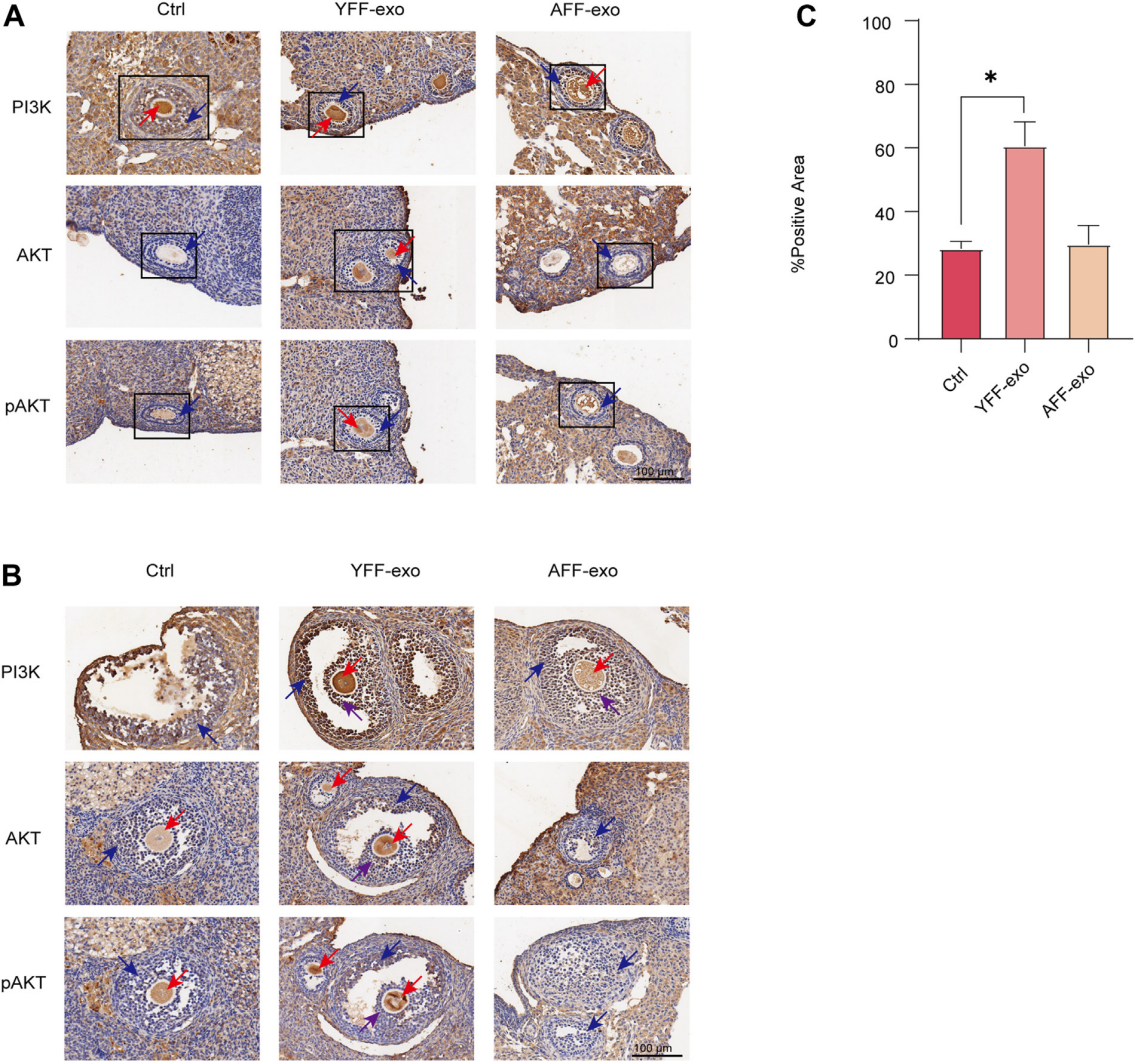
**Exosomes activates PI3K/AKT signaling to promote folliculogenesis.***A*, the expression levels of PI3K, AKT, and phosphorylated AKT (pAKT) in primary ovarian follicles collected after YFF-exo treatment were analyzed through immunohistochemical staining. The rectangular box encloses the primary follicle in each graph. The *red arrow* indicates the oocyte, whereas the *blue arrows* indicate the granulosa cells within the follicle. Adherent sections were subjected to staining with specific primary antibodies when feasible. Bar = 100 μm. *B*, the expression of PI3K, AKT, and pAKT in activated ovarian follicles obtained following YFF-exo treatment was assessed via immunohistochemical staining. The *red arrow* indicates the oocyte, whereas the *blue arrows* highlight the granulosa cells within the follicle. Additionally, the *purple arrow* denotes the presence of cumulus cells surrounding the oocyte. Bar = 100 μm. *C*, quantification of pAKT expression in the antral follicle between groups. The *p*-value is 0.207.

Immunohistochemical analysis was performed to assess the activation of the PI3K/AKT signaling pathway in the ovaries of aging and exosome-treated mice. In primary follicles, no significant differences in PI3K or pAKT expression were observed between the groups, except for increased AKT expression in the oocytes of YFF-exo-treated mice (Fig. 6*A*). As the follicles progressed to the antral stage, PI3K and AKT expression levels remained similar in the granulosa cells of the follicles (Fig. 6*B*). The expression of PI3K and AKT appeared higher in the YFF-exo group, but it could not be quantified due to the loss of oocytes in the aging and AFF-exo-treated groups. However, pAKT expression was notably higher in the follicles of the YFF-exo group compared to the AFF-exo group (Fig. 6*B*). Quantification of the IHC results confirmed that the increased expression of pAKT in the antral follicles after YFF-exo treatment was significantly higher than in the other groups (Fig. 6*C*). These results suggest that the application of YFF-exo may promote the activation of the PI3K/AKT signaling pathway, thereby enhancing follicular maturation after ovulation.

## Discussion

Previous data have shown that the fertility rate of women worldwide significantly decreases with age, and a predictive model suggests that the global decrease in the fertility rate will continue until 2100 (28, 29, 30). Aging has been suggested to be an important determinant of infertility (31). The follicular fluid, which surrounds the oocyte, has been shown to play a role in the quality and maturation of the oocyte (32). In this study, for the first time, we isolated exosomes from the follicular fluid of young and aging women and identified the proteins contained within them via label-free quantification (iBAQ).

Bioinformatics analysis revealed the differential expression of 69 proteins between the two groups. Further investigation of these proteins suggested that the immune system and metabolism are the two primary factors affecting the follicle environment, ultimately contributing to infertility. Previous studies have reported the upregulation of immune response proteins during pregnancy, illustrating the critical role of a healthy immune system in providing an environment conducive for embryo development (33, 34, 35). Importantly, inflammation, considered a hallmark of aging, has a detrimental effect on female fertility. A study revealed that as female mice age, their follicle count decreases, their percentage of intraovarian immune-associated cells increases, and their mRNA levels of inflammatory cytokines increase (36). Moreover, studies employing animal models have shown that depleting the inflammasome NLRP3 or the immune response to fibrotic collagen leads to the restoration of ovarian function in aging mice (37, 38). Based on our findings in the human population, aging individuals are more susceptible to pathogen invasion. Notably, the processes involving B-cell activation and maturation constitute a primary immune response in this context. This heightened inflammatory state observed in aging women suggested an abnormal ovarian environment, compromising oocyte maturation and follicle development.

Furthermore, metabolism is closely related to female fertility and is reported to decrease with age, as shown in previous studies (39, 40). Previous research has established a close link between metabolism and female fertility, with metabolic decline and disrupted DNA methylation identified as contributors to poor embryo quality (41). Although the role of carbon metabolism and its mediator folate in fertility has been investigated, a comprehensive understanding of the relationship between metabolism and aging-associated infertility is lacking. Recent studies have highlighted the regulatory role of tyrosine SHP2 phosphorylation in follicle development via PI3K/AKT signaling (42). Thus, our data suggested that DEPs related to carbon metabolism or tyrosine metabolism in the aging group might affect female fertility via exosomal protein regulation. Thus, attenuated metabolism in aging women is suggested to be another key factor that may contribute to infertility.

Our analysis revealed significant differences in several signaling pathways between the young and aging groups. Specifically, we observed variations in the activation of the Wnt and Hippo signaling pathways, as indicated by differentially expressed proteins (DEPs) in the exosomes from both groups. However, only a limited number of DEPs were found to be directly involved in these two pathways. In contrast, a substantial portion of the DEPs were associated with the PI3K/AKT signaling pathway, suggesting that this axis may serve as the predominant regulatory mechanism in FF-exosomes. Previous studies have demonstrated that depletion of Tsc2 in mice significantly reduces the maturation of primordial follicles, while further evidence supports the critical role of the PI3K/AKT pathway in folliculogenesis, with potential implications for fertility (43, 44). Our findings revealed notable differences in PI3K/AKT signaling between the young and aged groups, with reduced activation in the latter.

Subsequent *in vivo* experiments validated our proteomic results, revealing that exosomes from the aging group exhibited a diminished capacity to enhance ovarian function and failed to promote folliculogenesis, in contrast to exosomes from the young group. These results suggest that exosomes may play a pivotal role in mediating communication within the follicular microenvironment by regulating folliculogenesis via the PI3K/AKT signaling pathway.

Our investigation sought to characterize the proteomic profile of follicular fluid proteins associated with successful IVF outcomes, aiming to establish an age-related protein signature in FF-exosomes. We identified a total of 10 highly significant proteins. Among these proteins, FETUB is well known for its function associated with fertility (45). ENO1, an essential protein for mitochondrial function, has an unclear role in oocytes; However, studies have suggested that glycolysis regulated by ENO1 is important in granulosa cells, which in turn promotes follicle activation through mTOR signaling (46). Previous studies have suggested that deletion of HSP90B1 inhibits mitosis in mouse zygotes, and more recent literature indicates that its high expression in the cow follicular microenvironment is required for follicle maturation, although its role in humans remains unknown (47, 48). Additionally, C7, a component of the complement system, was not differentially expressed *in vivo*, but studies suggest it may contribute to oocyte maturation (49). Thus, these proteins were either verified in animal models or recently discovered to be associated with follicle maturation *in vivo*. However, the underlying molecular mechanisms still require further verification.

## Conclusion

This study identified distinct proteomic profiles of exosomes in the follicular fluid of young and aging patients. Exosomes from aging follicular fluid (AFF-exos) were associated with increased inflammation and increased susceptibility to pathogen invasion, triggering immune responses and influencing B-cell activation. The suppressed metabolic processes observed in aging women, together with immune activation, were found to contribute to the aging of the follicular microenvironment. Additionally, key proteins linked to impaired folliculogenesis in aging women were identified, resulting in reduced oocyte quality. Notably, the PI3K/AKT signaling pathway was shown to be regulated by exosomes. Differentially expressed protein (DEP) signatures associated with age-related infertility were detected in the follicular fluid proteome, providing novel insights into the impact of aging on female fertility and highlighting hydrolase activity as a potential therapeutic target for aging women experiencing IVF failure.

## Data Availability

The mass spectrometry proteomics data are available via MS-Viewer and ProteomeXchange. The MS-Viewer dataset can be accessed using the search key rwdy5skm0d at the following link: https://msviewer.ucsf.edu/prospector/cgi-bin/mssearch.cgi?report_title=MS-Viewer&search_key=rwdy5skm0d&search_name=msviewer.

The ProteomeXchange accession number for the dataset is PXD041139. Reviewer account details: Username: reviewer_pxd041139@ebi.ac.uk; Password: Znmwyh6q.

## Supplementary Data

This article contains supplementary data.

## Conflict of Interests

The authors declare that they have no conflicts of interest with the contents of this article.
