# Supplementary material for: Proteomic Analysis of Human Follicular Fluid-Derived Exosomes Reveals That Insufficient Folliculogenesis in Aging Women is Associated With Infertility
Source: Mol Cell Proteomics. 2025 Feb 28;24(4):100930. doi: 10.1016/j.mcpro.2025.100930 (PMC11994977; doi:10.1016/j.mcpro.2025.100930)
Supplement: Supplementary Figures [file mmc1.docx]

Supplementary Data for

**Proteomic analysis of human follicular fluid-derived exosomes reveals insufficient folliculogenesis in aging women associated with infertility**

Zhen Liu, Qilin Zhou, Jun Zan, Jingyan Tian, Yangzhuohan zhang, Fanggui Wu, Mengyuan Qu, Huan Zhao, Qianwen Peng, Shangjie Liu, Qianjun Chen, Endong Liu, Zhengdong Liao, Pengfei Zou, Lin Mei, Wen Wang, Sen Dong, Luo Niu, Shengda Wu, Liangge He, Xiaoyi Zhou, Yanbo Jin, Panpan Li, Sheng Yang*

*Corresponding author

Address correspondence to Prof. Sheng Yang, The Reproductive Medicine Center, The Third Affiliated Hospital of ShenZhen University, No. 47 Youyi Rd, Shenzhen, China. Tel.: +86 18617133989; E-mail: [tobyys2000@aliyun.com](mailto:tobyys2000@aliyun.com)

**Table of Contents**

**Supplementary Figures…………………………………………………………………………………..……….…S3**

Figure S1: Characterization of FF-exos isolated from young and aging women……………..S3

Figure S2： PCA cluster scatter plot of aging and young groups.………………………...…..S5

Figure S3：The distribution of abundance values among different samples.…………….......S6

Figure S4：Effect of follicular fluid exosomes on granulosa cells proliferation and viability...S7

Figure S5：Evaluation of ovary volume in the in vivo animal model……………………..…….S8

Figure S6：Verification of exosomal marker proteins and housekeeping protein…………….S9

Figure S7：Delivery of key proteins by exosomes vial tail vein injection…………………….S10

**Supplementary Figures**


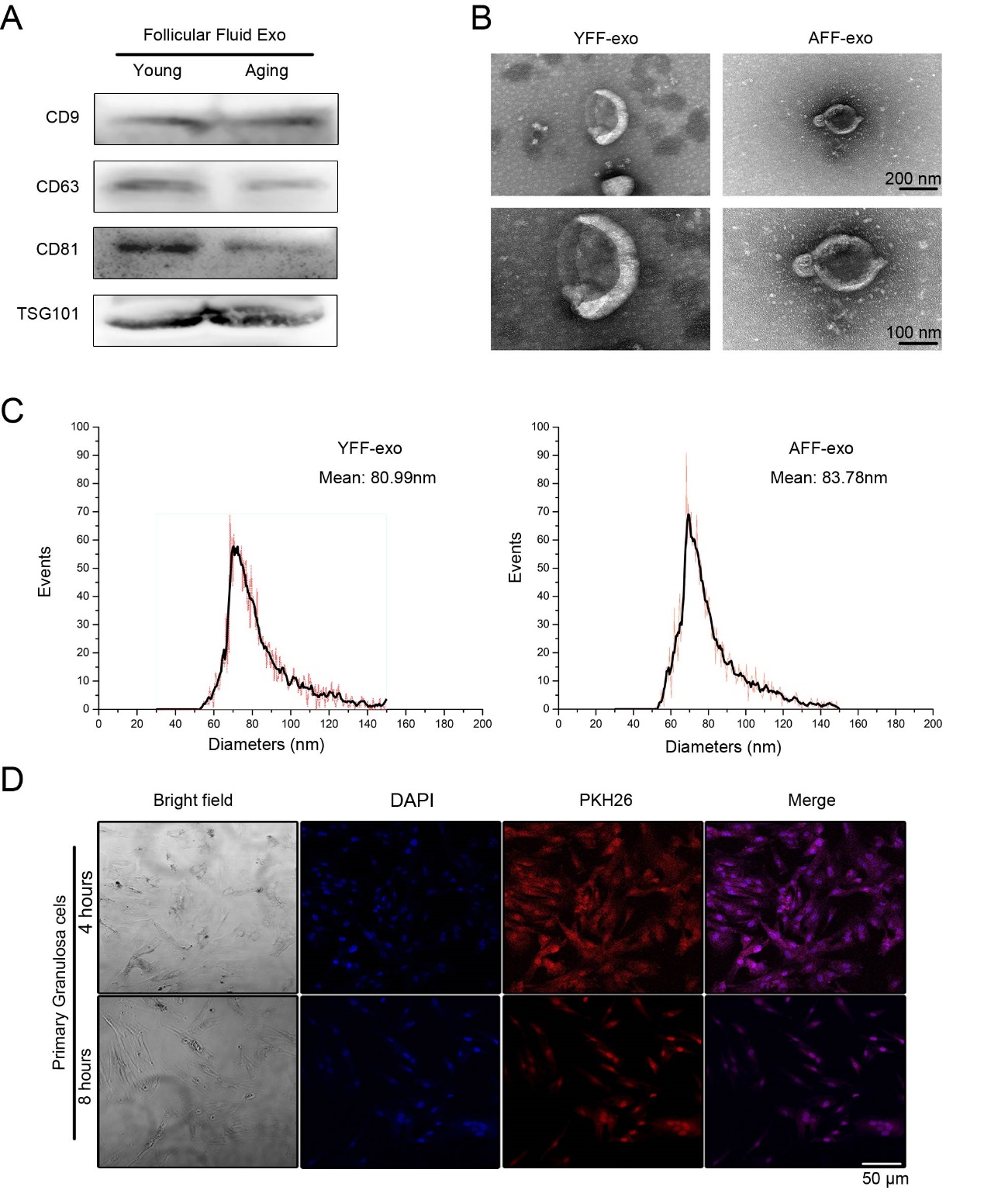


*Figure S1*

**Characterization of FF-exos isolated from young and aging women.** (A). Western blot analysis of exosomal proteins, including CD9, CD63, CD81, and TSG101, in follicular fluid. The experiments were repeated 3 times. (B) Morphological structure of follicular fluid-derived exosomes from the young and agingaged groups. The morphology was observed usingvia TEM. (C) The particle size distribution of FF-exos in the young and agingaged groups. The nanoparticle size was determined using NTA. (D) Uptake of FF-exos by primary granulosa cells. Fluorescence microscopy was appliedused to visualize the uptake of FF-exos by granulosa cells in the young and agingaged groups. The exosomes were labeled with PKH26 (shown in red), while the nuclei were labeled with DAPI (shown in blue). The experiments were repeated 3 times.


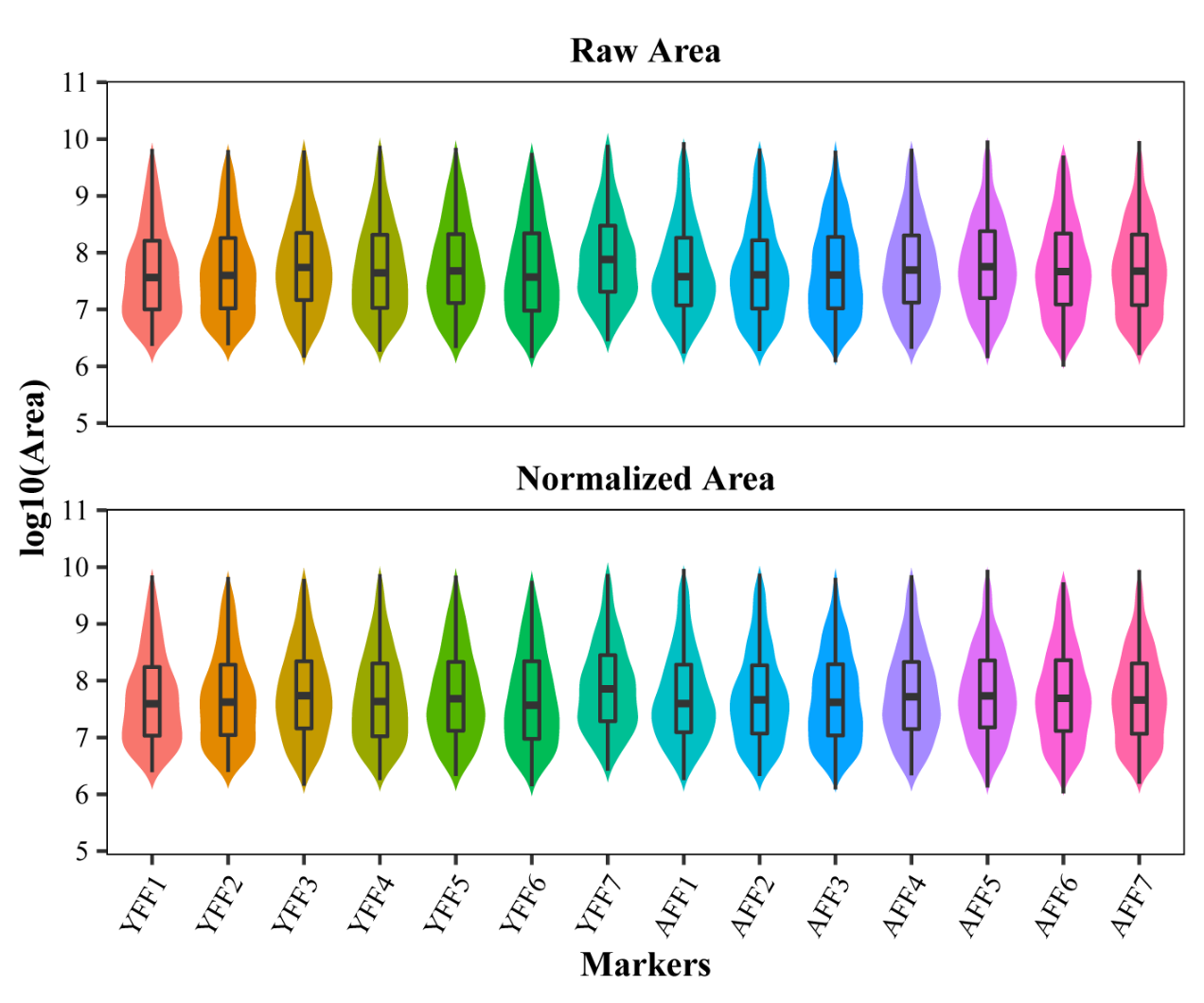


*Figure. S2*

**The distribution of abundance values among different samples.** The horizontal axis represents the sample names, and the vertical axis represents the log_10_ abundance values. The raw/normalized area represents the distribution plot of the original and normalized abundance values. The closer the box plots of biological replicates, the more similar the samples are.


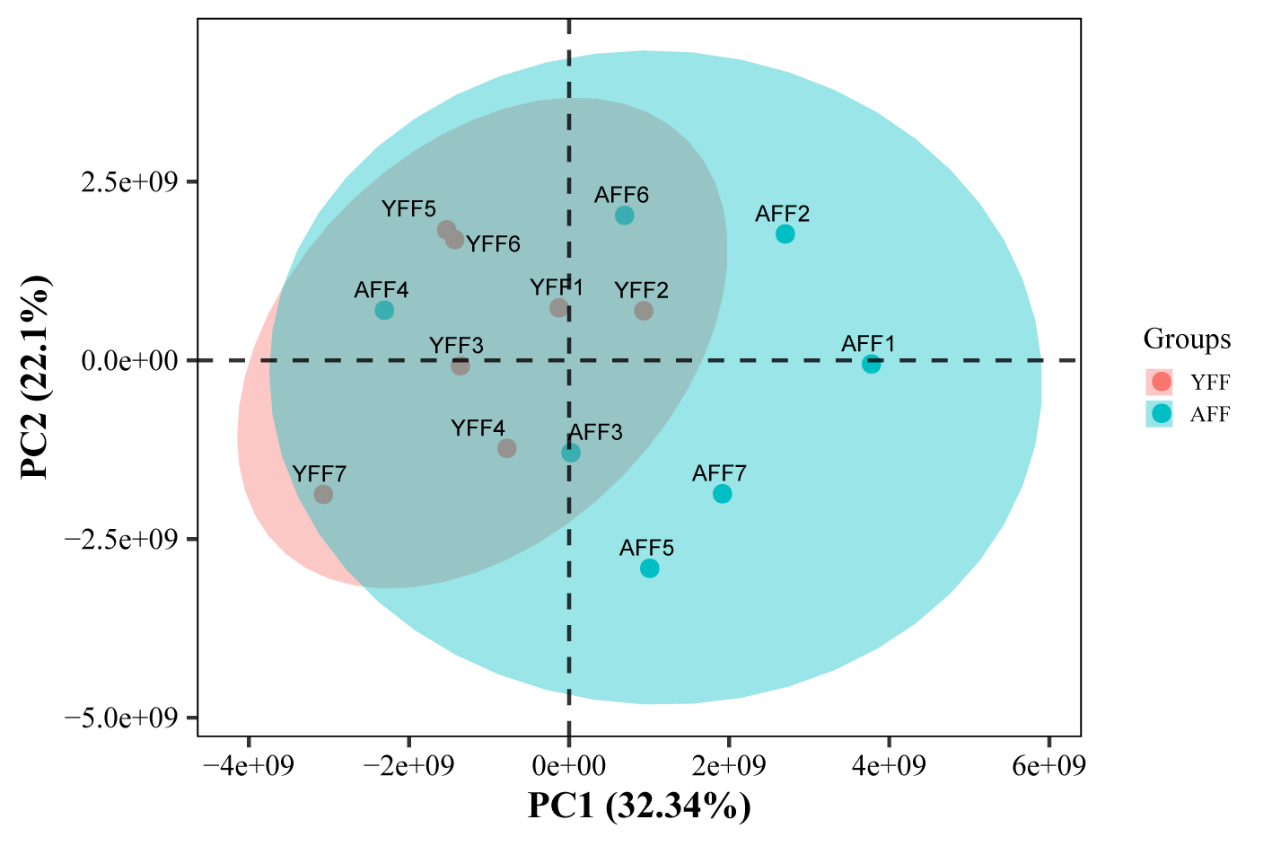


*Figure. S3*

**PCA cluster scatter plot of aging and young groups.** Better clustering between replicate samples indicates better quantitative reproducibility. PC1 and PC2 are represented on the x and y axes, respectively.


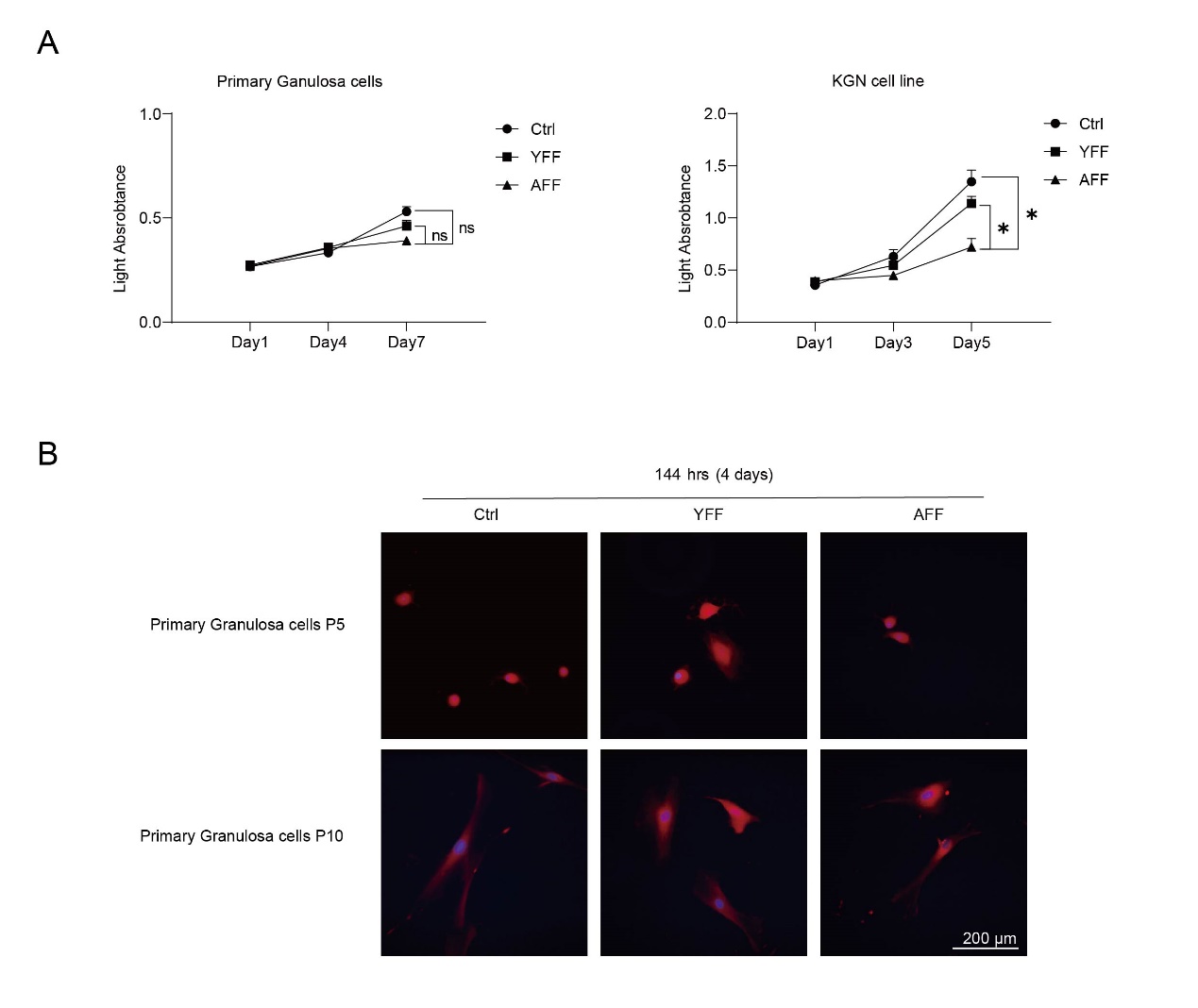


*Figure. S4*

**Effect of follicular fluid exosomes on granulosa cells proliferation and viability.** (A). CCK-8 Assay for evaluating cell proliferation in primary granulosa cells and KGN granulosa cell Lines. The y-axis represents light absorption measured at 450 nm. In primary granulosa cells, both *P-values* were found to be greater than 0.05. Within the KGN granulosa cell lines, the *P-value* comparing the Ctrl to the treatment with AFF was 0.0157, whereas the *P-value* comparing YFF to AFF was 0.038. (B). Immunofluorescent Staining of Mitochondria Using MitoTracker in Primary Granulosa Cells at Different Passages.


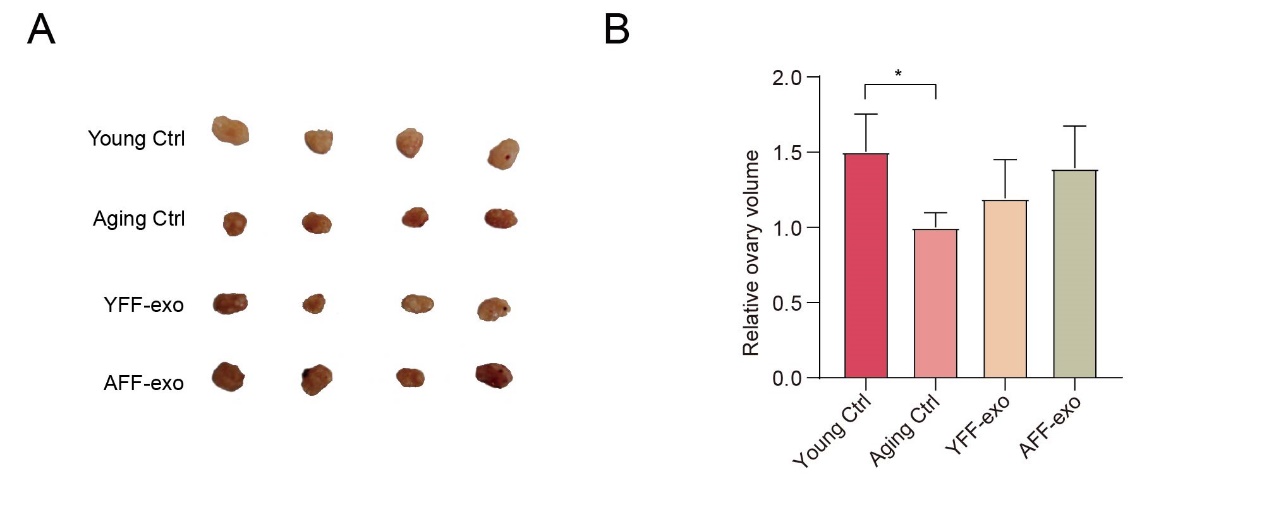


*Figure. S5*

**Evaluation of ovary volume in the in vivo animal model.** (A) Image showing the ovary from each group after sacrificing the mice (n=4 for each group). (B) Quantification of ovarian volume based on area measurements using ImageJ software. *P-value* = 0.0210 (n=4 for each group).


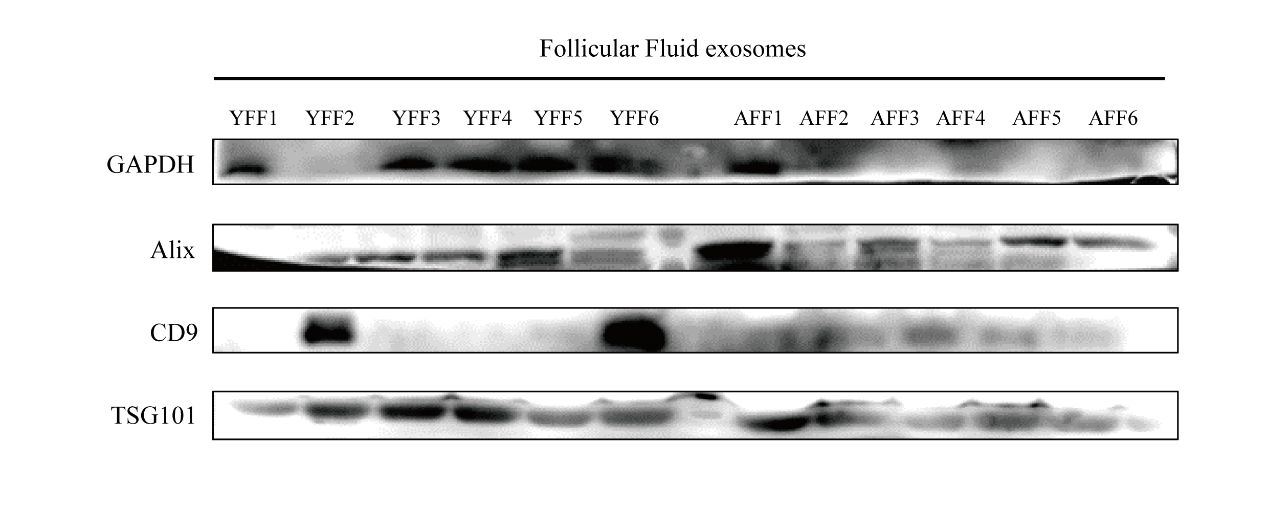


*Figure. S6*

**Verification of exosomal marker proteins and housekeeping protein.** Western Blot analysis of exosomal key signature proteins, including CD9, Alix, TSG101, and the housekeeping protein GAPDH, in YFF-exo and AFF-exo Samples (n=6 for Each Group).


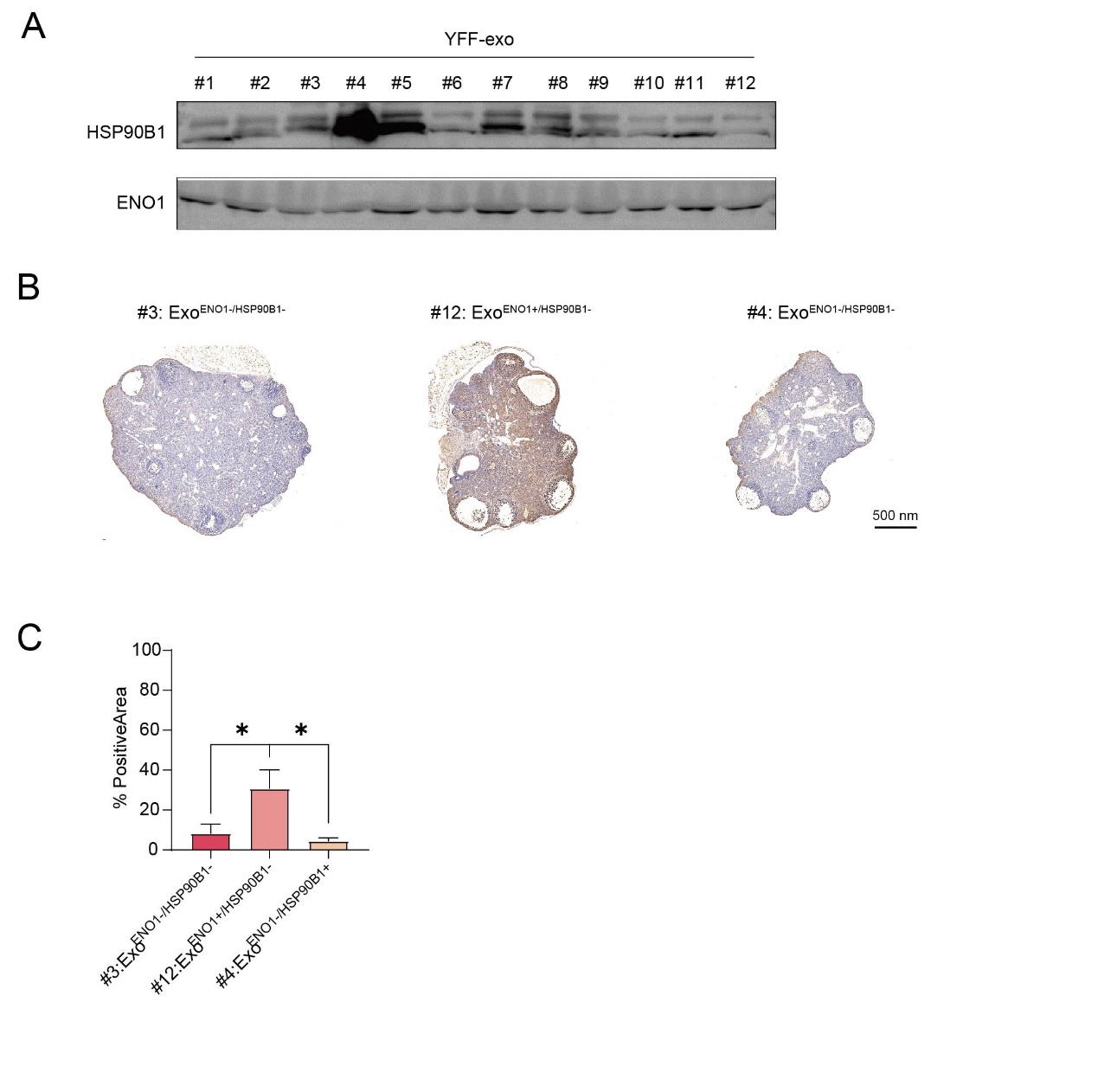


*Figure. S7*

**Delivery of key proteins by exosomes vial tail vein injection.** (A) Western blot analysis of exosomal proteins ENO1 and HSP90B1 in 12 samples. (B) IHC staining of ENO1 in ovaries from three groups (n=3 per group). (C) Quantification of ENO1 expression in these groups using ImageJ software. *P-values* = 0.337 and 0.379 for each (n=3 per group).
