# Supplementary material for: Proteomic Analysis of Human Follicular Fluid-Derived Exosomes Reveals That Insufficient Folliculogenesis in Aging Women is Associated With Infertility
Source: Mol Cell Proteomics. 2025 Feb 28;24(4):100930. doi: 10.1016/j.mcpro.2025.100930 (PMC11994977; doi:10.1016/j.mcpro.2025.100930)
Supplement: Supplementary Tables [file mmc2.docx]

Supplementary Data for

**Proteomic analysis of human follicular fluid-derived exosomes reveals insufficient folliculogenesis in aging women associated with infertility**

Zhen Liu, Qilin Zhou, Jun Zan, Jingyan Tian, Yangzhuohan zhang, Fanggui Wu, Mengyuan Qu, Huan Zhao, Qianwen Peng, Shangjie Liu, Qianjun Chen, Endong Liu, Zhengdong Liao, Pengfei Zou, Lin Mei, Wen Wang, Sen Dong, Luo Niu, Shengda Wu, Liangge He, Xiaoyi Zhou, Yanbo Jin, Panpan Li, Sheng Yang*

*Corresponding author

Address correspondence to Prof. Sheng Yang, The Reproductive Medicine Center, The Third Affiliated Hospital of ShenZhen University, No. 47 Youyi Rd, Shenzhen, China. Tel.: +86 18617133989; E-mail: [tobyys2000@aliyun.com](mailto:tobyys2000@aliyun.com)

**Table of Contents**

**Supplementary Tables………………………………………….…………………………….…S3**

Table S1: Quantification of identified proteins………………………...………………...………..S3

Table S2: Differentially expressed proteins in aging and young groups……………………..S12

Table S3: KEGG enrichment of differentially expressed proteins.……………\…….………..S14

Table S4 KEGG enrichment of differentially expressed proteins……………………….……..S20

**Supplementary Tables**

**Table S1: Quantification of identified proteins.**

| **Accession** | **GeneSymbol** | **Number of proteins** | **Peptides** | **Unique + razor sequence coverage [%]** | **AFF:YFF** |
| --- | --- | --- | --- | --- | --- |
| sp\|Q9BSK4\|FEM1A_HUMAN | FEM1A | 2 | 1 | 0.9 | 0 |
| sp\|A0A075B6H9\|LV469_HUMAN | IGLV4-69 | 1 | 1 | 8.4 | 0 |
| sp\|A0A075B6I0\|LV861_HUMAN | IGLV8-61 | 1 | 3 | 20.5 | 0.866 |
| sp\|A0A075B6I1\|LV460_HUMAN | IGLV4-60 | 1 | 1 | 8.3 | 0 |
| sp\|A0A075B6J9\|LV218_HUMAN | IGLV2-18 | 1 | 2 | 24.6 | 0 |
| sp\|A0A075B6K0\|LV316_HUMAN | IGLV3-16 | 1 | 4 | 7.8 | 0 |
| sp\|A0A075B6K2\|LV312_HUMAN | IGLV3-12 | 1 | 2 | 7 | 1.2 |
| sp\|A0A075B6K5\|LV39_HUMAN | IGLV3-9 | 2 | 4 | 30.4 | 0.927 |
| sp\|P01615\|KVD28_HUMAN | IGKV2D-28 | 2 | 3 | 29.2 | 1.256 |
| sp\|A0A075B6Q5\|HV364_HUMAN | IGHV3-64 | 1 | 1 | 9.3 | 0.948 |
| sp\|A0A075B6R2\|HV404_HUMAN | IGHV4-4 | 1 | 2 | 17.9 | 0 |
| sp\|A0A0C4DH68\|KV224_HUMAN | IGKV2-24 | 2 | 2 | 27.5 | 0.808 |
| sp\|A0A075B6S6\|KVD30_HUMAN | IGKV2D-30 | 2 | 3 | 25.8 | 0.836 |
| sp\|P0DSN7\|KVD37_HUMAN | IGKV1D-37 | 2 | 1 | 13.7 | 0 |
| sp\|A0A087WSY4\|HV432_HUMAN | IGHV4-30-2 | 1 | 2 | 17.8 | 0 |
| tr\|A0A0G2JQJ0\|A0A0G2JQJ0_HUMAN | IGKV1D-8 | 2 | 1 | 16.2 | 1.467 |
| sp\|P04433\|KV311_HUMAN | IGKV3-11 | 2 | 3 | 26.1 | 1.124 |
| sp\|A0A0A0MS15\|HV349_HUMAN | IGHV3-49 | 1 | 5 | 52.9 | 0.904 |
| sp\|A0A0A0MT36\|KVD21_HUMAN | IGKV6D-21 | 2 | 2 | 23.7 | 0 |
| sp\|A0A0B4J1U3\|LV136_HUMAN | IGLV1-36 | 1 | 1 | 13.7 | 0 |
| sp\|A0A0B4J1U7\|HV601_HUMAN | IGHV6-1 | 1 | 3 | 21.5 | 0.859 |
| sp\|A0A0B4J1V0\|HV315_HUMAN | IGHV3-15 | 1 | 2 | 20.2 | 0.772 |
| sp\|A0A0B4J1V2\|HV226_HUMAN | IGHV2-26 | 1 | 1 | 11.8 | 1.074 |
| sp\|A0A0B4J1X5\|HV374_HUMAN | IGHV3-74 | 3 | 3 | 22.2 | 0.872 |
| sp\|A0A0B4J1X8\|HV343_HUMAN | IGHV3-43 | 1 | 2 | 9.3 | 0 |
| sp\|A0A0B4J1Y8\|LV949_HUMAN | IGLV9-49 | 1 | 3 | 22 | 0 |
| sp\|P0DP09\|KV113_HUMAN | IGKV1-13 | 2 | 1 | 13.7 | 0 |
| sp\|A0A0B4J2H0\|HV69D_HUMAN | IGHV1-69D | 1 | 3 | 39.3 | 0.585 |
| sp\|A0A0C4DH24\|KV621_HUMAN | IGKV6-21 | 1 | 2 | 10.5 | 0 |
| sp\|A0A0C4DH25\|KVD20_HUMAN | IGKV3D-20 | 1 | 3 | 7.8 | 0 |
| sp\|A0A0C4DH29\|HV103_HUMAN | IGHV1-3 | 1 | 3 | 25.6 | 0.871 |
| sp\|A0A0C4DH31\|HV118_HUMAN | IGHV1-18 | 1 | 3 | 35.9 | 0.696 |
| sp\|A0A0C4DH33\|HV124_HUMAN | IGHV1-24 | 1 | 2 | 29.9 | 1.124 |
| sp\|A0A0C4DH34\|HV428_HUMAN | IGHV4-28 | 1 | 1 | 7.7 | 1.033 |
| sp\|A0A0C4DH35\|HV335_HUMAN | IGHV3-35 | 1 | 2 | 16.2 | 0 |
| sp\|A0A0C4DH36\|HV338_HUMAN | IGHV3-38 | 1 | 2 | 26.7 | 0.819 |
| sp\|A0A0C4DH38\|HV551_HUMAN | IGHV5-51 | 1 | 4 | 47 | 0.845 |
| sp\|A0A0C4DH67\|KV108_HUMAN | IGKV1-8 | 3 | 2 | 27 | 0.795 |
| sp\|A0A0C4DH72\|KV106_HUMAN | IGKV1-6 | 3 | 3 | 23.9 | 0.954 |
| sp\|A0A0G2JMI3\|HV692_HUMAN | IGHV1-69-2 | 1 | 1 | 17.1 | 0 |
| sp\|A0A0J9YVY3\|HV741_HUMAN | IGHV7-4-1 | 1 | 2 | 24.8 | 0 |
| sp\|A0A0J9YX35\|HV64D_HUMAN | IGHV3-64D | 1 | 1 | 9.4 | 0.929 |
| sp\|A0A0J9YXX1\|HV5X1_HUMAN | IGHV5-10-1 | 1 | 4 | 26.5 | 0 |
| tr\|A0A5H1ZRQ7\|A0A5H1ZRQ7_HUMAN | IGLC7 | 2 | 4 | 18.9 | 1.795 |
| sp\|A2NJV5\|KV229_HUMAN | IGKV2-29 | 1 | 4 | 40 | 1.686 |
| sp\|B9A064\|IGLL5_HUMAN | IGLL5 | 4 | 7 | 44.4 | 0.922 |
| sp\|O00264\|PGRC1_HUMAN | PGRMC1 | 1 | 2 | 11.3 | 1.773 |
| sp\|O00391\|QSOX1_HUMAN | QSOX1 | 1 | 7 | 11.5 | 1.121 |
| sp\|O00748\|EST2_HUMAN | CES2 | 3 | 5 | 11.8 | 0 |
| tr\|Q5JWQ4\|Q5JWQ4_HUMAN | NRP1 | 6 | 2 | 4.9 | 0 |
| sp\|O14791\|APOL1_HUMAN | APOL1 | 5 | 6 | 17.3 | 0.902 |
| tr\|Q5H919\|Q5H919_HUMAN | HTATSF1 | 3 | 1 | 7.2 | 0 |
| sp\|O43866\|CD5L_HUMAN | CD5L | 1 | 4 | 17.6 | 0 |
| tr\|U3KQK0\|U3KQK0_HUMAN | H2BC15 | 17 | 5 | 27.1 | 1.172 |
| tr\|A0A7P0TAB0\|A0A7P0TAB0_HUMAN | ERN1 | 2 | 1 | 1 | 1.129 |
| sp\|O75636\|FCN3_HUMAN | FCN3 | 1 | 5 | 24.4 | 0.985 |
| sp\|O75882\|ATRN_HUMAN | ATRN | 1 | 13 | 10.1 | 1.011 |
| sp\|O95445\|APOM_HUMAN | APOM | 2 | 3 | 32.4 | 0.907 |
| sp\|P00338\|LDHA_HUMAN | LDHA | 8 | 3 | 9.6 | 1.145 |
| sp\|P00450\|CERU_HUMAN | CP | 7 | 51 | 63.3 | 1.124 |
| sp\|P00488\|F13A_HUMAN | F13A1 | 4 | 4 | 7.4 | 0.686 |
| sp\|P00558\|PGK1_HUMAN | PGK1 | 2 | 2 | 7.9 | 0 |
| sp\|P00734\|THRB_HUMAN | F2 | 5 | 22 | 45.5 | 0.962 |
| tr\|A0A3B3ISR2\|A0A3B3ISR2_HUMAN | C1R | 8 | 17 | 32.2 | 1.085 |
| sp\|P00738\|HPT_HUMAN | HP | 12 | 30 | 60.8 | 1.094 |
| sp\|P00739\|HPTR_HUMAN | HPR | 2 | 21 | 17.2 | 1.025 |
| sp\|P00740\|FA9_HUMAN | F9 | 1 | 7 | 18 | 1.067 |
| sp\|P00742\|FA10_HUMAN | F10 | 1 | 3 | 9.4 | 1.119 |
| sp\|P00746\|CFAD_HUMAN | CFD | 2 | 3 | 22.1 | 0.974 |
| sp\|P00747\|PLMN_HUMAN | PLG | 8 | 30 | 53.5 | 0.899 |
| sp\|P00748\|FA12_HUMAN | F12 | 1 | 10 | 22.6 | 1.029 |
| tr\|E5RHP7\|E5RHP7_HUMAN | CA1 | 14 | 9 | 50.2 | 0.938 |
| sp\|P00918\|CAH2_HUMAN | CA2 | 3 | 3 | 15.8 | 1.254 |
| sp\|P01008\|ANT3_HUMAN | SERPINC1 | 3 | 21 | 43.1 | 1.029 |
| sp\|P01009\|A1AT_HUMAN | SERPINA1 | 8 | 34 | 70.3 | 1.302 |
| sp\|P01011\|AACT_HUMAN | SERPINA3 | 3 | 12 | 35 | 1.19 |
| tr\|A0A7P0TBH1\|A0A7P0TBH1_HUMAN | AGT | 7 | 13 | 36.4 | 1.892 |
| sp\|P01023\|A2MG_HUMAN | A2M | 6 | 75 | 65.6 |  |
| sp\|P01024\|CO3_HUMAN | C3 | 9 | 103 | 75.3 |  |
| sp\|P01031\|CO5_HUMAN | C5 | 3 | 52 | 40.6 | 0.992 |
| tr\|Q5H9A7\|Q5H9A7_HUMAN | TIMP1 | 5 | 3 | 27.3 | 1.19 |
| sp\|P01042\|KNG1_HUMAN | KNG1 | 1 | 19 | 32.5 | 1.114 |
| tr\|D6RD17\|D6RD17_HUMAN | JCHAIN | 4 | 4 | 28.7 | 0.507 |
| sp\|P01594\|KV133_HUMAN | IGKV1-33 | 2 | 1 | 13.7 | 1.291 |
| sp\|P04432\|KVD39_HUMAN | IGKV1D-39 | 2 | 3 | 15.4 | 0 |
| sp\|P01599\|KV117_HUMAN | IGKV1-17 | 1 | 3 | 12.8 | 0 |
| sp\|P01601\|KVD16_HUMAN | IGKV1D-16 | 1 | 1 | 13.7 | 0 |
| sp\|P01602\|KV105_HUMAN | IGKV1-5 | 1 | 3 | 24.8 | 0.814 |
| sp\|P01619\|KV320_HUMAN | IGKV3-20 | 1 | 3 | 27.6 | 1.024 |
| sp\|P01624\|KV315_HUMAN | IGKV3-15 | 1 | 2 | 20.9 | 1.014 |
| sp\|P01700\|LV147_HUMAN | IGLV1-47 | 2 | 2 | 18.8 | 0.765 |
| sp\|P01701\|LV151_HUMAN | IGLV1-51 | 1 | 2 | 7.7 | 0.839 |
| sp\|P01703\|LV140_HUMAN | IGLV1-40 | 1 | 1 | 25.4 | 0 |
| sp\|P01706\|LV211_HUMAN | IGLV2-11 | 1 | 2 | 37 | 0 |
| sp\|P01714\|LV319_HUMAN | IGLV3-19 | 1 | 2 | 18.8 | 0.779 |
| sp\|P01717\|LV325_HUMAN | IGLV3-25 | 1 | 5 | 36.6 | 0.686 |
| sp\|P01721\|LV657_HUMAN | IGLV6-57 | 1 | 3 | 35.9 | 0.843 |
| sp\|P01742\|HV169_HUMAN | IGHV1-69 | 1 | 3 | 11.1 | 1.026 |
| sp\|P01743\|HV146_HUMAN | IGHV1-46 | 1 | 3 | 25.6 | 1.065 |
| sp\|P01764\|HV323_HUMAN | IGHV3-23 | 1 | 3 | 18.8 | 0 |
| sp\|P01766\|HV313_HUMAN | IGHV3-13 | 1 | 2 | 9.5 | 0 |
| sp\|P01767\|HV353_HUMAN | IGHV3-53 | 1 | 3 | 18.1 | 0 |
| sp\|P0DP03\|HVC05_HUMAN | IGHV3-30-5 | 2 | 4 | 47.9 | 0.931 |
| sp\|P01780\|HV307_HUMAN | IGHV3-7 | 6 | 3 | 7.7 | 0.797 |
| sp\|P01782\|HV309_HUMAN | IGHV3-9 | 2 | 4 | 53.4 | 0.753 |
| sp\|P01834\|IGKC_HUMAN | IGKC | 2 | 7 | 80.4 | 1.024 |
| tr\|A0A0A0MS08\|A0A0A0MS08_HUMAN | IGHG1 | 3 | 17 | 54.1 | 0.969 |
| sp\|P01859\|IGHG2_HUMAN | IGHG2 | 2 | 14 | 31 | 1.025 |
| tr\|A0A4W9A917\|A0A4W9A917_HUMAN | IGHG3 | 3 | 17 | 40.1 | 1.378 |
| sp\|P01861\|IGHG4_HUMAN | IGHG4 | 4 | 16 | 39.1 | 2.391 |
| sp\|P01871\|IGHM_HUMAN | IGHM | 1 | 17 | 44.8 | 0.812 |
| sp\|P01876\|IGHA1_HUMAN | IGHA1 | 2 | 12 | 52.4 | 1.022 |
| sp\|P01877\|IGHA2_HUMAN | IGHA2 | 2 | 9 | 11.2 | 1.168 |
| sp\|P01880\|IGHD_HUMAN | IGHD | 2 | 7 | 28.9 | 20.366 |
| sp\|P02042\|HBD_HUMAN | HBD | 3 | 13 | 57.8 | 1.026 |
| tr\|A0A3B3ISV3\|A0A3B3ISV3_HUMAN | COL4A1 | 2 | 2 | 8 | 0 |
| sp\|P02647\|APOA1_HUMAN | APOA1 | 4 | 27 | 76.8 | 1.04 |
| sp\|P02649\|APOE_HUMAN | APOE | 6 | 15 | 54.3 | 0.994 |
| sp\|P02652\|APOA2_HUMAN | APOA2 | 5 | 7 | 64 | 0.868 |
| tr\|K7ERI9\|K7ERI9_HUMAN | APOC1 | 6 | 4 | 35.1 | 1.092 |
| sp\|P02656\|APOC3_HUMAN | APOC3 | 2 | 4 | 53.5 | 1.379 |
| sp\|P02671\|FIBA_HUMAN | FGA | 4 | 37 | 44.8 | 1.075 |
| sp\|P02675\|FIBB_HUMAN | FGB | 4 | 27 | 62.1 | 1.094 |
| sp\|P02679\|FIBG_HUMAN | FGG | 3 | 24 | 50.3 | 1.099 |
| sp\|P02730\|B3AT_HUMAN | SLC4A1 | 2 | 7 | 9.2 | 0 |
| sp\|P02741\|CRP_HUMAN | CRP | 3 | 5 | 21 | 2.064 |
| sp\|P02743\|SAMP_HUMAN | APCS | 1 | 7 | 30 | 0.845 |
| sp\|P02745\|C1QA_HUMAN | C1QA | 2 | 3 | 12.2 | 1.701 |
| tr\|A0A0A0MSV6\|A0A0A0MSV6_HUMAN | C1QB | 3 | 3 | 18.9 | 1.072 |
| sp\|P02747\|C1QC_HUMAN | C1QC | 1 | 4 | 21.2 | 0.924 |
| sp\|P02748\|CO9_HUMAN | C9 | 6 | 17 | 36 | 1.552 |
| sp\|P02749\|APOH_HUMAN | APOH | 6 | 12 | 38.8 | 1.051 |
| sp\|P02750\|A2GL_HUMAN | LRG1 | 1 | 10 | 37.8 | 1.429 |
| sp\|P02751\|FINC_HUMAN | FN1 | 3 | 62 | 37.7 | 1.086 |
| tr\|Q5VY30\|Q5VY30_HUMAN | RBP4 | 2 | 7 | 48.7 | 1.172 |
| sp\|P02760\|AMBP_HUMAN | AMBP | 5 | 13 | 51.4 | 0.973 |
| sp\|P02763\|A1AG1_HUMAN | ORM1 | 1 | 9 | 44.8 | 1.383 |
| sp\|P02765\|FETUA_HUMAN | AHSG | 4 | 6 | 27.8 | 1.248 |
| tr\|A0A087WT59\|A0A087WT59_HUMAN | TTR | 2 | 9 | 88.7 | 1.093 |
| sp\|P02774\|VTDB_HUMAN | GC | 8 | 20 | 36.1 | 1.324 |
| sp\|P02786\|TFR1_HUMAN | TFRC | 3 | 8 | 14.6 | 0.913 |
| sp\|P02787\|TRFE_HUMAN | TF | 9 | 50 | 67.3 | 1.093 |
| sp\|P02790\|HEMO_HUMAN | HPX | 2 | 20 | 54.8 | 1.085 |
| sp\|P03951\|FA11_HUMAN | F11 | 3 | 4 | 6.1 | 0.817 |
| sp\|P03952\|KLKB1_HUMAN | KLKB1 | 5 | 18 | 29.9 | 0.852 |
| sp\|P04003\|C4BPA_HUMAN | C4BPA | 1 | 7 | 14.2 | 0.986 |
| sp\|P04004\|VTNC_HUMAN | VTN | 5 | 13 | 30.3 | 1.165 |
| sp\|P04040\|CATA_HUMAN | CAT | 1 | 16 | 41.2 | 0.901 |
| tr\|H7BYX9\|H7BYX9_HUMAN | PROC | 3 | 2 | 11.9 | 1.252 |
| sp\|P04075\|ALDOA_HUMAN | ALDOA | 9 | 7 | 26.9 | 0.877 |
| sp\|P04114\|APOB_HUMAN | APOB | 2 | 2 | 0.7 | 0 |
| sp\|P04180\|LCAT_HUMAN | LCAT | 4 | 4 | 10.2 | 0.839 |
| sp\|P04196\|HRG_HUMAN | HRG | 1 | 16 | 35.4 | 0.801 |
| sp\|P04217\|A1BG_HUMAN | A1BG | 2 | 13 | 45.5 | 1.031 |
| tr\|I3L145\|I3L145_HUMAN | SHBG | 16 | 13 | 60.8 | 2.185 |
| sp\|P04406\|G3P_HUMAN | GAPDH | 2 | 7 | 32.8 | 1.654 |
| sp\|P04430\|KV116_HUMAN | IGKV1-16 | 1 | 2 | 26.5 | 0.754 |
| tr\|F8WF32\|F8WF32_HUMAN | RPN1 | 2 | 2 | 26.4 | 0 |
| tr\|A0A0U1RRH7\|A0A0U1RRH7_HUMAN | -- | 21 | 2 | 16.5 | 1.045 |
| sp\|P05090\|APOD_HUMAN | APOD | 4 | 6 | 34.4 | 1.046 |
| tr\|A0A1W2PQT5\|A0A1W2PQT5_HUMAN | CYP17A1 | 5 | 4 | 10.6 | 0.964 |
| sp\|P05111\|INHA_HUMAN | INHA | 1 | 3 | 11.2 | 0 |
| sp\|P05121\|PAI1_HUMAN | SERPINE1 | 1 | 2 | 7.5 | 0 |
| sp\|P05154\|IPSP_HUMAN | SERPINA5 | 6 | 10 | 29.1 | 0.869 |
| tr\|A0A7I2V2D2\|A0A7I2V2D2_HUMAN | SERPING1 | 10 | 14 | 28.2 | 1.061 |
| tr\|G3XAM2\|G3XAM2_HUMAN | CFI | 7 | 13 | 27.3 | 1.168 |
| sp\|P05160\|F13B_HUMAN | F13B | 1 | 3 | 4.4 | 0.927 |
| sp\|P05543\|THBG_HUMAN | SERPINA7 | 3 | 13 | 40.7 | 1.314 |
| sp\|P05546\|HEP2_HUMAN | SERPIND1 | 3 | 14 | 36.5 | 1.327 |
| tr\|A0A087WYX9\|A0A087WYX9_HUMAN | COL5A2 | 2 | 2 | 3.8 | 0 |
| sp\|P06276\|CHLE_HUMAN | BCHE | 5 | 8 | 23.1 | 0.839 |
| sp\|P06312\|KV401_HUMAN | IGKV4-1 | 1 | 4 | 29.8 | 0.896 |
| sp\|P06331\|HV434_HUMAN | IGHV4-34 | 7 | 3 | 37.4 | 0.904 |
| sp\|P06681\|CO2_HUMAN | C2 | 9 | 23 | 8.8 | 1.001 |
| sp\|P06727\|APOA4_HUMAN | APOA4 | 1 | 28 | 63.4 | 1.28 |
| sp\|P06733\|ENOA_HUMAN | ENO1 | 17 | 5 | 18.4 | 1.36 |
| sp\|P06858\|LIPL_HUMAN | LPL | 4 | 9 | 22.5 | 1.038 |
| sp\|P07093\|GDN_HUMAN | SERPINE2 | 3 | 17 | 52.8 | 0.87 |
| sp\|P07099\|HYEP_HUMAN | EPHX1 | 2 | 4 | 16.5 | 1.27 |
| tr\|A8MW50\|A8MW50_HUMAN | LDHB | 6 | 2 | 6.9 | 0 |
| tr\|G5E9F8\|G5E9F8_HUMAN | PROS1 | 6 | 9 | 18 | 1.014 |
| tr\|A0A7P0TA71\|A0A7P0TA71_HUMAN | P4HB | 32 | 5 | 11.7 | 2.015 |
| tr\|H0YMW4\|H0YMW4_HUMAN | ANXA2 | 20 | 4 | 13.1 | 1.435 |
| sp\|P07357\|CO8A_HUMAN | C8A | 1 | 12 | 31.5 | 0.901 |
| tr\|F5GY80\|F5GY80_HUMAN | C8B | 3 | 20 | 52.6 | 0.899 |
| sp\|P07360\|CO8G_HUMAN | C8G | 2 | 7 | 44.1 | 1.059 |
| sp\|P08238\|HS90B_HUMAN | HSP90AB1 | 4 | 2 | 3.6 | 1.338 |
| sp\|P08133\|ANXA6_HUMAN | ANXA6 | 10 | 10 | 20.5 | 1.413 |
| sp\|P08185\|CBG_HUMAN | SERPINA6 | 3 | 10 | 35.3 | 1.242 |
| sp\|P09210\|GSTA2_HUMAN | GSTA2 | 6 | 4 | 22.1 | 0 |
| tr\|D6RFL4\|D6RFL4_HUMAN | CD14 | 2 | 2 | 18.3 | 1.077 |
| sp\|P08572\|CO4A2_HUMAN | COL4A2 | 4 | 3 | 3 | 0.954 |
| sp\|P08603\|CFAH_HUMAN | CFH | 3 | 49 | 48.6 | 0.837 |
| sp\|P08697\|A2AP_HUMAN | SERPINF2 | 8 | 19 | 52.5 | 1.076 |
| sp\|P08758\|ANXA5_HUMAN | ANXA5 | 4 | 3 | 11.9 | 0 |
| sp\|P09172\|DOPO_HUMAN | DBH | 1 | 2 | 4.5 | 1.399 |
| tr\|A0A0A0MTB9\|A0A0A0MTB9_HUMAN | RBP1 | 4 | 2 | 23.1 | 0 |
| sp\|P09486\|SPRC_HUMAN | SPARC | 3 | 2 | 7.3 | 1.335 |
| sp\|P09871\|C1S_HUMAN | C1S | 7 | 17 | 29.9 | 1.021 |
| sp\|P0C0L4\|CO4A_HUMAN | C4A | 3 | 80 | 4.4 | 1.203 |
| sp\|P0C0L5\|CO4B_HUMAN | C4B | 4 | 80 | 55.1 | 1.148 |
| sp\|P0CG48\|UBC_HUMAN | UBC | 23 | 3 | 35.5 | 1.316 |
| sp\|P0DJI8\|SAA1_HUMAN | SAA1 | 2 | 4 | 28.7 | 0 |
| sp\|P0DOY2\|IGLC2_HUMAN | IGLC2 | 3 | 6 | 27.4 | 0.899 |
| sp\|P0DP08\|HVD82_HUMAN | IGHV4-38-2 | 1 | 3 | 17.9 | 0 |
| sp\|P10620\|MGST1_HUMAN | MGST1 | 4 | 2 | 18.7 | 0 |
| sp\|P10643\|CO7_HUMAN | C7 | 3 | 19 | 30.5 | 1.243 |
| tr\|A0A7I2YQK6\|A0A7I2YQK6_HUMAN | HSPD1 | 13 | 2 | 5 | 0 |
| sp\|P10909\|CLUS_HUMAN | CLU | 10 | 15 | 35.2 | 0.863 |
| sp\|P11021\|BIP_HUMAN | HSPA5 | 3 | 9 | 19.4 | 1.818 |
| sp\|P11047\|LAMC1_HUMAN | LAMC1 | 1 | 6 | 5 | 1.259 |
| sp\|P11226\|MBL2_HUMAN | MBL2 | 1 | 4 | 20.6 | 1.025 |
| sp\|P11511\|CP19A_HUMAN | CYP19A1 | 9 | 7 | 17.7 | 0.585 |
| tr\|H3BRJ9\|H3BRJ9_HUMAN | CETP | 2 | 3 | 8.4 | 1.058 |
| sp\|P12109\|CO6A1_HUMAN | COL6A1 | 3 | 4 | 6.8 | 1.18 |
| sp\|P13611\|CSPG2_HUMAN | VCAN | 4 | 11 | 4.5 | 0.76 |
| sp\|P13671\|CO6_HUMAN | C6 | 2 | 17 | 24.5 | 0.913 |
| sp\|P13716\|HEM2_HUMAN | ALAD | 2 | 3 | 13.9 | 0.528 |
| tr\|A0A7P0TAT8\|A0A7P0TAT8_HUMAN | HSP90B1 | 17 | 10 | 15.4 | 1.837 |
| sp\|P15090\|FABP4_HUMAN | FABP4 | 1 | 1 | 15.2 | 0 |
| sp\|P15169\|CBPN_HUMAN | CPN1 | 4 | 12 | 37.1 | 0.99 |
| sp\|P15309\|PPAP_HUMAN | ACP3 | 3 | 7 | 26.9 | 0.915 |
| sp\|P15814\|IGLL1_HUMAN | IGLL1 | 1 | 1 | 7 | 2.628 |
| tr\|H0Y4R2\|H0Y4R2_HUMAN | POR | 3 | 3 | 6.6 | 0 |
| sp\|P48741\|HSP77_HUMAN | HSPA7 | 11 | 2 | 6.5 | 0 |
| tr\|C9JMX4\|C9JMX4_HUMAN | IGFBP3 | 6 | 3 | 25 | 1.08 |
| sp\|P18428\|LBP_HUMAN | LBP | 1 | 6 | 17.5 | 1.625 |
| sp\|P19652\|A1AG2_HUMAN | ORM2 | 1 | 9 | 31.3 | 1.126 |
| tr\|Q5T985\|Q5T985_HUMAN | ITIH2 | 5 | 25 | 36.7 | 1.02 |
| sp\|P19827\|ITIH1_HUMAN | ITIH1 | 7 | 30 | 49.7 | 0.994 |
| sp\|P19883\|FST_HUMAN | FST | 3 | 2 | 8.1 | 0 |
| sp\|P20742\|PZP_HUMAN | PZP | 2 | 36 | 26.5 | 2.651 |
| tr\|A0A7P0NMY4\|A0A7P0NMY4_HUMAN | FLNA | 7 | 2 | 2 | 0 |
| tr\|A0A140T8Z8\|A0A140T8Z8_HUMAN | TNXB | 15 | 8 | 4.7 | 0.966 |
| tr\|A0A087X1J7\|A0A087X1J7_HUMAN | GPX3 | 5 | 4 | 24.4 | 0.964 |
| sp\|P22792\|CPN2_HUMAN | CPN2 | 1 | 10 | 25.1 | 0.884 |
| sp\|P22891\|PROZ_HUMAN | PROZ | 1 | 2 | 5.5 | 0 |
| sp\|P23083\|HV102_HUMAN | IGHV1-2 | 1 | 4 | 54.7 | 1.344 |
| tr\|H7BZ97\|H7BZ97_HUMAN | ITGA6 | 2 | 1 | 5 | 0 |
| tr\|A0A7P0Z497\|A0A7P0Z497_HUMAN | PPIB | 4 | 3 | 19.3 | 1.328 |
| sp\|P24593\|IBP5_HUMAN | IGFBP5 | 2 | 2 | 8.1 | 1.009 |
| sp\|P25311\|ZA2G_HUMAN | AZGP1 | 3 | 11 | 48 | 1.227 |
| sp\|P25391\|LAMA1_HUMAN | LAMA1 | 1 | 4 | 2 | 0.934 |
| tr\|A0A024RA52\|A0A024RA52_HUMAN | PSMA2 | 6 | 2 | 17.1 | 0 |
| sp\|P26022\|PTX3_HUMAN | PTX3 | 1 | 3 | 8.9 | 0 |
| sp\|P26439\|3BHS2_HUMAN | HSD3B2 | 4 | 6 | 26.9 | 2.953 |
| sp\|P26927\|HGFL_HUMAN | MST1 | 4 | 5 | 8.9 | 1.21 |
| sp\|P27105\|STOM_HUMAN | STOM | 2 | 3 | 17.7 | 0 |
| sp\|P27169\|PON1_HUMAN | PON1 | 2 | 12 | 61.4 | 0.84 |
| tr\|A0A7P0T861\|A0A7P0T861_HUMAN | CALR | 3 | 3 | 10.1 | 0 |
| sp\|P27918\|PROP_HUMAN | CFP | 2 | 3 | 6.4 | 0.847 |
| sp\|P28070\|PSB4_HUMAN | PSMB4 | 1 | 1 | 8.3 | 0 |
| sp\|P28074\|PSB5_HUMAN | PSMB5 | 2 | 2 | 8.4 | 0 |
| tr\|A0A0A0MQV1\|A0A0A0MQV1_HUMAN | HSD11B1 | 2 | 1 | 4 | 0 |
| sp\|P29622\|KAIN_HUMAN | SERPINA4 | 1 | 15 | 47.8 | 1.102 |
| sp\|P30043\|BLVRB_HUMAN | BLVRB | 5 | 4 | 32.5 | 0 |
| sp\|P30101\|PDIA3_HUMAN | PDIA3 | 2 | 8 | 20.6 | 1.526 |
| sp\|P32119\|PRDX2_HUMAN | PRDX2 | 5 | 4 | 19.7 | 0.863 |
| sp\|P33908\|MA1A1_HUMAN | MAN1A1 | 1 | 5 | 8.4 | 0.951 |
| tr\|A0A2R8Y7M3\|A0A2R8Y7M3_HUMAN | RDX | 6 | 2 | 3.4 | 0 |
| tr\|E7ES19\|E7ES19_HUMAN | THBS4 | 6 | 5 | 8 | 0.766 |
| sp\|P35579\|MYH9_HUMAN | MYH9 | 1 | 1 | 1 | 0 |
| sp\|P35858\|ALS_HUMAN | IGFALS | 1 | 17 | 37.5 | 1.124 |
| sp\|P36955\|PEDF_HUMAN | SERPINF1 | 11 | 15 | 44.5 | 1.225 |
| tr\|V9GYE7\|V9GYE7_HUMAN | CFHR2 | 7 | 5 | 8.7 | 0 |
| sp\|P43251\|BTD_HUMAN | BTD | 10 | 9 | 20.8 | 1.143 |
| sp\|P43652\|AFAM_HUMAN | AFM | 3 | 14 | 23.7 | 1.002 |
| sp\|P47972\|NPTX2_HUMAN | NPTX2 | 1 | 3 | 11.8 | 0.726 |
| tr\|F8W876\|F8W876_HUMAN | MASP1 | 2 | 2 | 7.1 | 0.893 |
| tr\|A0A087WXQ8\|A0A087WXQ8_HUMAN | PSMB3 | 4 | 1 | 16.8 | 0 |
| tr\|A0A182DWH7\|A0A182DWH7_HUMAN | SELENOP | 2 | 3 | 10 | 0.901 |
| sp\|P51884\|LUM_HUMAN | LUM | 3 | 8 | 30.5 | 1.296 |
| sp\|P55058\|PLTP_HUMAN | PLTP | 1 | 5 | 12.6 | 0.874 |
| tr\|A0A7P0Z4C6\|A0A7P0Z4C6_HUMAN | VCP | 11 | 8 | 16.1 | 1.267 |
| sp\|P55268\|LAMB2_HUMAN | LAMB2 | 1 | 4 | 3.8 | 0 |
| sp\|P60709\|ACTB_HUMAN | ACTB | 27 | 11 | 42.9 | 1.296 |
| tr\|F8VV32\|F8VV32_HUMAN | LYZ | 3 | 3 | 27.9 | 1.283 |
| sp\|P61769\|B2MG_HUMAN | B2M | 3 | 3 | 35.3 | 1.189 |
| sp\|P62805\|H4_HUMAN | H4C1 | 1 | 4 | 38.8 | 0.9 |
| tr\|E7EX29\|E7EX29_HUMAN | YWHAZ | 17 | 2 | 7.7 | 0.901 |
| tr\|A0A7I2V3H3\|A0A7I2V3H3_HUMAN | EEF1A1 | 15 | 3 | 9 | 1.18 |
| tr\|F5H5D3\|F5H5D3_HUMAN | TUBA1C | 25 | 3 | 6.9 | 0 |
| sp\|P68871\|HBB_HUMAN | HBB | 5 | 14 | 95.2 | 7.401 |
| sp\|P69892\|HBG2_HUMAN | HBG2 | 2 | 8 | 58.5 | 1.709 |
| sp\|P69905\|HBA_HUMAN | HBA1 | 2 | 10 | 91.5 | 7.451 |
| sp\|P78539\|SRPX_HUMAN | SRPX | 1 | 2 | 5.6 | 0 |
| tr\|A0A804HLA0\|A0A804HLA0_HUMAN | PHEX | 2 | 2 | 5.8 | 0 |
| sp\|P80108\|PHLD_HUMAN | GPLD1 | 1 | 10 | 16.8 | 0.856 |
| sp\|P80748\|LV321_HUMAN | IGLV3-21 | 1 | 4 | 25.6 | 0.881 |
| sp\|P83110\|HTRA3_HUMAN | HTRA3 | 3 | 2 | 5.1 | 0 |
| sp\|P98066\|TSG6_HUMAN | TNFAIP6 | 1 | 2 | 9.7 | 0 |
| sp\|P98160\|PGBM_HUMAN | HSPG2 | 5 | 136 | 51.5 | 0.842 |
| tr\|A0A087WYK9\|A0A087WYK9_HUMAN | CFHR3 | 3 | 3 | 10.3 | 1.096 |
| tr\|B1AKG0\|B1AKG0_HUMAN | CFHR1 | 3 | 7 | 17.3 | 1.013 |
| tr\|C9JEK3\|C9JEK3_HUMAN | CALD1 | 5 | 1 | 14.4 | 0 |
| sp\|Q06033\|ITIH3_HUMAN | ITIH3 | 5 | 13 | 20.2 | 0.961 |
| sp\|Q08380\|LG3BP_HUMAN | LGALS3BP | 10 | 15 | 30.8 | 0.759 |
| sp\|Q08431\|MFGM_HUMAN | MFGE8 | 2 | 3 | 9 | 0 |
| sp\|Q08830\|FGL1_HUMAN | FGL1 | 1 | 2 | 5.8 | 0 |
| tr\|A0A0U1RQV3\|A0A0U1RQV3_HUMAN | EFEMP1 | 2 | 2 | 12.9 | 1.496 |
| tr\|B7Z683\|B7Z683_HUMAN | ABR | 10 | 2 | 3 | 0.8 |
| sp\|Q13103\|SPP24_HUMAN | SPP2 | 2 | 2 | 11.4 | 0.916 |
| tr\|C9J4H5\|C9J4H5_HUMAN | SEMA3F | 3 | 1 | 5.5 | 0 |
| sp\|Q13790\|APOF_HUMAN | APOF | 1 | 1 | 4.3 | 1.026 |
| sp\|Q14520\|HABP2_HUMAN | HABP2 | 1 | 8 | 10.9 | 1.204 |
| sp\|Q14624\|ITIH4_HUMAN | ITIH4 | 5 | 31 | 41.3 | 1.062 |
| sp\|Q14697\|GANAB_HUMAN | GANAB | 3 | 4 | 6.1 | 1.842 |
| sp\|Q15084\|PDIA6_HUMAN | PDIA6 | 1 | 1 | 3 | 0 |
| sp\|Q15392\|DHC24_HUMAN | DHCR24 | 4 | 4 | 9.7 | 0 |
| sp\|Q15485\|FCN2_HUMAN | FCN2 | 1 | 4 | 16.6 | 0.697 |
| tr\|H0Y8L3\|H0Y8L3_HUMAN | TGFBI | 5 | 4 | 13.7 | 1.09 |
| sp\|Q15848\|ADIPO_HUMAN | ADIPOQ | 1 | 1 | 6.1 | 1.116 |
| sp\|Q16610\|ECM1_HUMAN | ECM1 | 1 | 9 | 28.3 | 0.975 |
| sp\|Q16658\|FSCN1_HUMAN | FSCN1 | 1 | 2 | 4.5 | 1.287 |
| sp\|Q16850\|CP51A_HUMAN | CYP51A1 | 1 | 3 | 6.5 | 0 |
| sp\|Q16853\|AOC3_HUMAN | AOC3 | 1 | 2 | 2.9 | 0 |
| sp\|Q3T8J9\|GON4L_HUMAN | GON4L | 1 | 2 | 0.9 | 1.33 |
| sp\|Q5SZK8\|FREM2_HUMAN | FREM2 | 1 | 3 | 1.4 | 0 |
| sp\|Q6P387\|CP046_HUMAN | C16orf46 | 1 | 1 | 2.5 | 0.885 |
| sp\|Q6ZRK6\|CCD73_HUMAN | CCDC73 | 1 | 1 | 1.4 | 0 |
| sp\|Q8IWY4\|SCUB1_HUMAN | SCUBE1 | 5 | 3 | 4.1 | 0.6 |
| tr\|A0A0C4DG40\|A0A0C4DG40_HUMAN | SYNE1 | 9 | 2 | 0.3 | 0 |
| tr\|A0A2R8Y5P9\|A0A2R8Y5P9_HUMAN | SHROOM3 | 2 | 3 | 1.4 | 1.465 |
| tr\|A0A7P0T9B3\|A0A7P0T9B3_HUMAN | SCARB1 | 8 | 2 | 7.2 | 0 |
| tr\|A0A7I2YQQ1\|A0A7I2YQQ1_HUMAN | SCFD1 | 19 | 2 | 5.2 | 0 |
| sp\|Q8WWA0\|ITLN1_HUMAN | ITLN1 | 1 | 2 | 9.3 | 0 |
| tr\|A0A0A0MRA3\|A0A0A0MRA3_HUMAN | TTN | 3 | 2 | 0.1 | 0.891 |
| tr\|A0A7I2YQQ3\|A0A7I2YQQ3_HUMAN | GGH | 4 | 3 | 13.1 | 0 |
| sp\|Q96HR9\|REEP6_HUMAN | REEP6 | 1 | 1 | 6.2 | 0 |
| sp\|Q96IY4\|CBPB2_HUMAN | CPB2 | 4 | 7 | 25.3 | 1.07 |
| sp\|Q96KN2\|CNDP1_HUMAN | CNDP1 | 2 | 9 | 24.3 | 1.041 |
| sp\|Q96PD5\|PGRP2_HUMAN | PGLYRP2 | 4 | 12 | 37.7 | 0.927 |
| sp\|Q99584\|S10AD_HUMAN | S100A13 | 1 | 2 | 23.5 | 0 |
| sp\|Q9BSW2\|EFC4B_HUMAN | CRACR2A | 2 | 2 | 5.1 | 0 |
| tr\|Q5T4F6\|Q5T4F6_HUMAN | CRTAC1 | 3 | 3 | 6.9 | 0 |
| sp\|Q9NZP8\|C1RL_HUMAN | C1RL | 8 | 5 | 9.7 | 1.02 |
| sp\|Q9P2B2\|FPRP_HUMAN | PTGFRN | 1 | 2 | 3.2 | 0 |
| tr\|C9IYF5\|C9IYF5_HUMAN | EXTL2 | 4 | 1 | 16.2 | 0 |
| tr\|E9PG08\|E9PG08_HUMAN | FETUB | 4 | 2 | 5 | 1.369 |
| sp\|Q9UHG3\|PCYOX_HUMAN | PCYOX1 | 5 | 2 | 5.5 | 0.834 |
| sp\|Q9UJC3\|HOOK1_HUMAN | HOOK1 | 1 | 2 | 4 | 0 |
| sp\|Q9UK55\|ZPI_HUMAN | SERPINA10 | 4 | 6 | 18 | 1.537 |
| tr\|A0A075B7B8\|A0A075B7B8_HUMAN | IGHV3OR16-12 | 1 | 2 | 9.4 | 0 |
| tr\|A0A075B7D0\|A0A075B7D0_HUMAN | IGHV1OR15-1 | 1 | 1 | 10.3 | 0 |
| tr\|A0A096LPE2\|A0A096LPE2_HUMAN | SAA2-SAA4 | 6 | 4 | 23.6 | 0.885 |
| tr\|A0A0G2JMB2\|A0A0G2JMB2_HUMAN | IGHA2 | 1 | 9 | 13.2 | 1.323 |
| tr\|A0A0G2JRQ6\|A0A0G2JRQ6_HUMAN | -- | 1 | 3 | 26.5 | 0.698 |
| tr\|A0A0G2JSC0\|A0A0G2JSC0_HUMAN | IGLV5-45 | 3 | 2 | 15.4 | 0.907 |
| tr\|A0A0J9YY99\|A0A0J9YY99_HUMAN | -- | 1 | 4 | 7.7 | 0 |
| tr\|A0A4W8ZXM2\|A0A4W8ZXM2_HUMAN | IGHV3-72 | 2 | 5 | 72.3 | 0.808 |
| tr\|B1AHL2\|B1AHL2_HUMAN | FBLN1 | 1 | 5 | 8 | 1.263 |
| tr\|B4E1Z4\|B4E1Z4_HUMAN | -- | 18 | 46 | 40.9 | 1.072 |
| tr\|C9IZP8\|C9IZP8_HUMAN | C1S | 1 | 1 | 16.5 | 0 |
| tr\|G3V1N2\|G3V1N2_HUMAN | HBA2 | 1 | 8 | 7.3 | 0 |
| tr\|G3V3A0\|G3V3A0_HUMAN | SERPINA3 | 1 | 8 | 5.9 | 0 |
| tr\|K7ER74\|K7ER74_HUMAN | APOC4-APOC2 | 6 | 4 | 27 | 1.387 |

**Table S2: Differentially expressed proteins in aging and young groups.**

| **Gene Symbol** | **Accession** | **YFF:AFF** | **YFF:AFF adj. PVal** | **YFF:AFF State** |
| --- | --- | --- | --- | --- |
| IGHD | sp\|P01880\|IGHD_HUMAN | 20.366 | 0.010801 | down |
| HSD3B2 | sp\|P26439\|3BHS2_HUMAN | 2.953 | 0.026272 | down |
| PZP | sp\|P20742\|PZP_HUMAN | 2.651 | 0.00012 | down |
| IGHG4 | sp\|P01861\|IGHG4_HUMAN | 2.391 | 1.17E-12 | down |
| SHBG | tr\|I3L145\|I3L145_HUMAN | 2.185 | 1.9E-09 | down |
| AGT | tr\|A0A7P0TBH1\|A0A7P0TBH1_HUMAN | 1.892 | 9.39E-17 | down |
| GANAB | sp\|Q14697\|GANAB_HUMAN | 1.842 | 0.000336 | down |
| HSP90B1 | tr\|A0A7P0TAT8\|A0A7P0TAT8_HUMAN | 1.837 | 0.01245 | down |
| HSPA5 | sp\|P11021\|BIP_HUMAN | 1.818 | 3.17E-06 | down |
| IGLC7 | tr\|A0A5H1ZRQ7\|A0A5H1ZRQ7_HUMAN | 1.795 | 1.35E-09 | down |
| PGRMC1 | sp\|O00264\|PGRC1_HUMAN | 1.773 | 0.025225 | down |
| C1QA | sp\|P02745\|C1QA_HUMAN | 1.701 | 0.001591 | down |
| IGKV2-29 | sp\|A2NJV5\|KV229_HUMAN | 1.686 | 2.42E-05 | down |
| GAPDH | sp\|P04406\|G3P_HUMAN | 1.654 | 0.02054 | down |
| LBP | sp\|P18428\|LBP_HUMAN | 1.625 | 1.64E-09 | down |
| C9 | sp\|P02748\|CO9_HUMAN | 1.552 | 2.88E-07 | down |
| SERPINA10 | sp\|Q9UK55\|ZPI_HUMAN | 1.537 | 2.79E-08 | down |
| PDIA3 | sp\|P30101\|PDIA3_HUMAN | 1.526 | 0.011601 | down |
| IGKV1D-8 | tr\|A0A0G2JQJ0\|A0A0G2JQJ0_HUMAN | 1.467 | 0.001308 | down |
| SHROOM3 | tr\|A0A2R8Y5P9\|A0A2R8Y5P9_HUMAN | 1.465 | 1.17E-12 | down |
| LRG1 | sp\|P02750\|A2GL_HUMAN | 1.429 | 3.7E-12 | down |
| DBH | sp\|P09172\|DOPO_HUMAN | 1.399 | 0.000292 | down |
| APOC4-APOC2 | tr\|K7ER74\|K7ER74_HUMAN | 1.387 | 2.03E-09 | down |
| ORM1 | sp\|P02763\|A1AG1_HUMAN | 1.383 | 0.00157 | down |
| APOC3 | sp\|P02656\|APOC3_HUMAN | 1.379 | 3.55E-06 | down |
| IGHG3 | tr\|A0A4W9A917\|A0A4W9A917_HUMAN | 1.378 | 0.048638 | down |
| FETUB | tr\|E9PG08\|E9PG08_HUMAN | 1.369 | 7.7E-06 | down |
| ENO1 | sp\|P06733\|ENOA_HUMAN | 1.36 | 0.010557 | down |
| SERPIND1 | sp\|P05546\|HEP2_HUMAN | 1.327 | 2E-08 | down |
| GC | sp\|P02774\|VTDB_HUMAN | 1.324 | 4.35E-20 | down |
| UBC | sp\|P0CG48\|UBC_HUMAN | 1.316 | 3.08E-06 | down |
| SERPINA7 | sp\|P05543\|THBG_HUMAN | 1.314 | 6.93E-12 | down |
| SERPINA1 | sp\|P01009\|A1AT_HUMAN | 1.302 | 2.46E-13 | down |
| ACTB | sp\|P60709\|ACTB_HUMAN | 1.296 | 0.023768 | down |
| LUM | sp\|P51884\|LUM_HUMAN | 1.296 | 1.96E-07 | down |
| IGKV1-33 | sp\|P01594\|KV133_HUMAN | 1.291 | 7.16E-07 | down |
| LYZ | tr\|F8VV32\|F8VV32_HUMAN | 1.283 | 0.001143 | down |
| APOA4 | sp\|P06727\|APOA4_HUMAN | 1.28 | 7.27E-05 | down |
| FBLN1 | tr\|B1AHL2\|B1AHL2_HUMAN | 1.263 | 2.64E-07 | down |
| PROC | tr\|H7BYX9\|H7BYX9_HUMAN | 1.252 | 1.18E-05 | down |
| AHSG | sp\|P02765\|FETUA_HUMAN | 1.248 | 1.63E-10 | down |
| C7 | sp\|P10643\|CO7_HUMAN | 1.243 | 0.026367 | down |
| SERPINA6 | sp\|P08185\|CBG_HUMAN | 1.242 | 0.003559 | down |
| AZGP1 | sp\|P25311\|ZA2G_HUMAN | 1.227 | 3.47E-06 | down |
| SERPINF1 | sp\|P36955\|PEDF_HUMAN | 1.225 | 7.14E-07 | down |
| MST1 | sp\|P26927\|HGFL_HUMAN | 1.21 | 5.79E-06 | down |
| IGHV3-38 | sp\|A0A0C4DH36\|HV338_HUMAN | 0.819 | 0.016858 | up |
| IGKV1-5 | sp\|P01602\|KV105_HUMAN | 0.814 | 6.33E-05 | up |
| IGHM | sp\|P01871\|IGHM_HUMAN | 0.812 | 0.000147 | up |
| IGHV3-72 | tr\|A0A4W8ZXM2\|A0A4W8ZXM2_HUMAN | 0.808 | 2.35E-07 | up |
| IGKV2-24 | sp\|A0A0C4DH68\|KV224_HUMAN | 0.808 | 0.029796 | up |
| HRG | sp\|P04196\|HRG_HUMAN | 0.801 | 1.84E-08 | up |
| IGHV3-7 | sp\|P01780\|HV307_HUMAN | 0.797 | 1.11E-05 | up |
| IGKV1-8 | sp\|A0A0C4DH67\|KV108_HUMAN | 0.795 | 2.86E-09 | up |
| IGLV3-19 | sp\|P01714\|LV319_HUMAN | 0.779 | 1.94E-08 | up |
| IGHV3-15 | sp\|A0A0B4J1V0\|HV315_HUMAN | 0.772 | 0.029947 | up |
| THBS4 | tr\|E7ES19\|E7ES19_HUMAN | 0.766 | 2E-05 | up |
| IGLV1-47 | sp\|P01700\|LV147_HUMAN | 0.765 | 3.66E-07 | up |
| VCAN | sp\|P13611\|CSPG2_HUMAN | 0.76 | 1.1E-05 | up |
| LGALS3BP | sp\|Q08380\|LG3BP_HUMAN | 0.759 | 8.39E-11 | up |
| IGKV1-16 | sp\|P04430\|KV116_HUMAN | 0.754 | 0.005666 | up |
| IGHV3-9 | sp\|P01782\|HV309_HUMAN | 0.753 | 1.03E-10 | up |
| -- | tr\|A0A0G2JRQ6\|A0A0G2JRQ6_HUMAN | 0.698 | 0.008079 | up |
| FCN2 | sp\|Q15485\|FCN2_HUMAN | 0.697 | 7.5E-05 | up |
| IGHV1-18 | sp\|A0A0C4DH31\|HV118_HUMAN | 0.696 | 1.35E-07 | up |
| IGLV3-25 | sp\|P01717\|LV325_HUMAN | 0.686 | 8.12E-10 | up |
| F13A1 | sp\|P00488\|F13A_HUMAN | 0.686 | 3.98E-06 | up |
| IGHV1-69D | sp\|A0A0B4J2H0\|HV69D_HUMAN | 0.585 | 4.51E-08 | up |
| JCHAIN | tr\|D6RD17\|D6RD17_HUMAN | 0.507 | 2.42E-14 | up |

**Table S3: GO function analysis of differentially expressed proteins.**

| \| **Gene Symbol** \| **YFF:AFF State** \| **GO function (Biological Process)** \| \| --- \| --- \| --- \| \| IGHD \| down \| immune system process(GO:0002376);metabolic process(GO:0008152);cellular process(GO:0009987);biological process involved in interspecies interaction between organisms(GO:0044419);response to stimulus(GO:0050896);localization(GO:0051179);biological regulation(GO:0065007) \| \| HSD3B2 \| down \| metabolic process(GO:0008152);cellular process(GO:0009987);biological regulation(GO:0065007) \| \| PZP \| down \| metabolic process(GO:0008152);cellular process(GO:0009987);reproductive process(GO:0022414);multicellular organismal process(GO:0032501);developmental process(GO:0032502);response to stimulus(GO:0050896);localization(GO:0051179);multi-organism process(GO:0051704);biological regulation(GO:0065007) \| \| IGHG4 \| down \| immune system process(GO:0002376);metabolic process(GO:0008152);cellular process(GO:0009987);biological process involved in interspecies interaction between organisms(GO:0044419);response to stimulus(GO:0050896);biological regulation(GO:0065007) \| \| SHBG \| down \| -- \| \| AGT \| down \| behavior(GO:0007610);metabolic process(GO:0008152);cellular process(GO:0009987);reproductive process(GO:0022414);multicellular organismal process(GO:0032501);developmental process(GO:0032502);growth(GO:0040007);response to stimulus(GO:0050896);multi-organism process(GO:0051704);biological regulation(GO:0065007) \| \| GANAB \| down \| metabolic process(GO:0008152);cellular process(GO:0009987) \| \| HSP90B1 \| down \| metabolic process(GO:0008152);cellular process(GO:0009987);response to stimulus(GO:0050896);localization(GO:0051179);biological regulation(GO:0065007) \| \| HSPA5 \| down \| metabolic process(GO:0008152);cellular process(GO:0009987);biological adhesion(GO:0022610);developmental process(GO:0032502);response to stimulus(GO:0050896);localization(GO:0051179);biological regulation(GO:0065007) \| \| IGLC7 \| down \| immune system process(GO:0002376);metabolic process(GO:0008152);cellular process(GO:0009987);biological process involved in interspecies interaction between organisms(GO:0044419);response to stimulus(GO:0050896);localization(GO:0051179);biological regulation(GO:0065007) \| \| PGRMC1 \| down \| cellular process(GO:0009987);reproductive process(GO:0022414) \| \| C1QA \| down \| immune system process(GO:0002376);metabolic process(GO:0008152);cellular process(GO:0009987);signaling(GO:0023052);biological process involved in interspecies interaction between organisms(GO:0044419);response to stimulus(GO:0050896);biological regulation(GO:0065007) \| \| IGKV2-29 \| down \| immune system process(GO:0002376);metabolic process(GO:0008152);cellular process(GO:0009987);response to stimulus(GO:0050896);localization(GO:0051179);biological regulation(GO:0065007) \| \| GAPDH \| down \| metabolic process(GO:0008152);cellular process(GO:0009987);response to stimulus(GO:0050896);biological regulation(GO:0065007) \| \| LBP \| down \| immune system process(GO:0002376);cellular process(GO:0009987);developmental process(GO:0032502);locomotion(GO:0040011);biological process involved in interspecies interaction between organisms(GO:0044419);response to stimulus(GO:0050896);localization(GO:0051179);biological regulation(GO:0065007) \| \| C9 \| down \| immune system process(GO:0002376);cellular process(GO:0009987);multicellular organismal process(GO:0032501);biological process involved in interspecies interaction between organisms(GO:0044419);response to stimulus(GO:0050896);biological regulation(GO:0065007) \| \| SERPINA10 \| down \| metabolic process(GO:0008152);cellular process(GO:0009987);multicellular organismal process(GO:0032501);developmental process(GO:0032502);growth(GO:0040007);biological regulation(GO:0065007) \| \| PDIA3 \| down \| immune system process(GO:0002376);metabolic process(GO:0008152);cellular process(GO:0009987);rhythmic process(GO:0048511);response to stimulus(GO:0050896);localization(GO:0051179);biological regulation(GO:0065007) \| \| IGKV1D-8 \| down \| -- \| \| SHROOM3 \| down \| cellular process(GO:0009987);multicellular organismal process(GO:0032501);developmental process(GO:0032502);pigmentation(GO:0043473);response to stimulus(GO:0050896);localization(GO:0051179);biological regulation(GO:0065007) \| \| LRG1 \| down \| cellular process(GO:0009987);developmental process(GO:0032502);biological regulation(GO:0065007) \| \| DBH \| down \| immune system process(GO:0002376);behavior(GO:0007610);metabolic process(GO:0008152);cellular process(GO:0009987);reproductive process(GO:0022414);signaling(GO:0023052);multicellular organismal process(GO:0032501);locomotion(GO:0040011);response to stimulus(GO:0050896);biological regulation(GO:0065007) \| \| APOC4-APOC2 \| down \| metabolic process(GO:0008152);cellular process(GO:0009987);multicellular organismal process(GO:0032501);localization(GO:0051179);biological regulation(GO:0065007) \| \| ORM1 \| down \| cellular process(GO:0009987);response to stimulus(GO:0050896);localization(GO:0051179);biological regulation(GO:0065007) \| \| APOC3 \| down \| metabolic process(GO:0008152);cellular process(GO:0009987);multicellular organismal process(GO:0032501);response to stimulus(GO:0050896);localization(GO:0051179);biological regulation(GO:0065007) \| \| IGHG3 \| down \| immune system process(GO:0002376);metabolic process(GO:0008152);cellular process(GO:0009987);biological process involved in interspecies interaction between organisms(GO:0044419);response to stimulus(GO:0050896);biological regulation(GO:0065007) \| \| FETUB \| down \| cellular process(GO:0009987);reproductive process(GO:0022414);biological regulation(GO:0065007) \| \| ENO1 \| down \| metabolic process(GO:0008152);cellular process(GO:0009987);biological adhesion(GO:0022610);multicellular organismal process(GO:0032501);developmental process(GO:0032502);biological process involved in interspecies interaction between organisms(GO:0044419);response to stimulus(GO:0050896);biological regulation(GO:0065007) \| \| SERPIND1 \| down \| metabolic process(GO:0008152);cellular process(GO:0009987);multicellular organismal process(GO:0032501);locomotion(GO:0040011);biological regulation(GO:0065007) \| \| GC \| down \| metabolic process(GO:0008152);cellular process(GO:0009987);reproductive process(GO:0022414);multicellular organismal process(GO:0032501);developmental process(GO:0032502);response to stimulus(GO:0050896);localization(GO:0051179);multi-organism process(GO:0051704);biological regulation(GO:0065007) \| \| UBC \| down \| immune system process(GO:0002376);metabolic process(GO:0008152);cellular process(GO:0009987);viral process(GO:0016032);reproductive process(GO:0022414);developmental process(GO:0032502);response to stimulus(GO:0050896);localization(GO:0051179);multi-organism process(GO:0051704);biological regulation(GO:0065007) \| \| SERPINA7 \| down \| localization(GO:0051179);biological regulation(GO:0065007) \| \| SERPINA1 \| down \| cellular process(GO:0009987);multicellular organismal process(GO:0032501);response to stimulus(GO:0050896);localization(GO:0051179);biological regulation(GO:0065007) \| \| ACTB \| down \| immune system process(GO:0002376);cellular process(GO:0009987);biological adhesion(GO:0022610);developmental process(GO:0032502);rhythmic process(GO:0048511);response to stimulus(GO:0050896);biological regulation(GO:0065007) \| \| LUM \| down \| metabolic process(GO:0008152);cellular process(GO:0009987);multicellular organismal process(GO:0032501);developmental process(GO:0032502);response to stimulus(GO:0050896);biological regulation(GO:0065007) \| \| IGKV1-33 \| down \| immune system process(GO:0002376);metabolic process(GO:0008152);cellular process(GO:0009987);biological process involved in interspecies interaction between organisms(GO:0044419);response to stimulus(GO:0050896);localization(GO:0051179);biological regulation(GO:0065007) \| \| LYZ \| down \| immune system process(GO:0002376);metabolic process(GO:0008152);cellular process(GO:0009987);multicellular organismal process(GO:0032501);biological process involved in interspecies interaction between organisms(GO:0044419);response to stimulus(GO:0050896);biological regulation(GO:0065007) \| \| APOA4 \| down \| immune system process(GO:0002376);metabolic process(GO:0008152);cellular process(GO:0009987);biological adhesion(GO:0022610);multicellular organismal process(GO:0032501);developmental process(GO:0032502);biological process involved in interspecies interaction between organisms(GO:0044419);response to stimulus(GO:0050896);localization(GO:0051179);biological regulation(GO:0065007);detoxification(GO:0098754) \| \| FBLN1 \| down \| metabolic process(GO:0008152);cellular process(GO:0009987);reproductive process(GO:0022414);response to stimulus(GO:0050896);biological regulation(GO:0065007) \| \| PROC \| down \| immune system process(GO:0002376);metabolic process(GO:0008152);cellular process(GO:0009987);locomotion(GO:0040011);localization(GO:0051179);biological regulation(GO:0065007) \| \| AHSG \| down \| immune system process(GO:0002376);cellular process(GO:0009987);developmental process(GO:0032502);response to stimulus(GO:0050896);localization(GO:0051179);biological regulation(GO:0065007) \| \| C7 \| down \| immune system process(GO:0002376);cellular process(GO:0009987);biological process involved in interspecies interaction between organisms(GO:0044419);response to stimulus(GO:0050896);biological regulation(GO:0065007) \| \| SERPINA6 \| down \| metabolic process(GO:0008152);localization(GO:0051179);biological regulation(GO:0065007) \| \| AZGP1 \| down \| immune system process(GO:0002376);metabolic process(GO:0008152);cellular process(GO:0009987);biological adhesion(GO:0022610);response to stimulus(GO:0050896);localization(GO:0051179);biological regulation(GO:0065007) \| \| SERPINF1 \| down \| behavior(GO:0007610);cellular process(GO:0009987);reproductive process(GO:0022414);multicellular organismal process(GO:0032501);developmental process(GO:0032502);rhythmic process(GO:0048511);response to stimulus(GO:0050896);biological regulation(GO:0065007) \| \| MST1 \| down \| metabolic process(GO:0008152);cellular process(GO:0009987);reproductive process(GO:0022414);multicellular organismal process(GO:0032501);developmental process(GO:0032502);growth(GO:0040007);locomotion(GO:0040011);biological regulation(GO:0065007) \| \| IGHV3-38 \| up \| immune system process(GO:0002376);metabolic process(GO:0008152);cellular process(GO:0009987);biological process involved in interspecies interaction between organisms(GO:0044419);response to stimulus(GO:0050896);localization(GO:0051179);biological regulation(GO:0065007) \| \| IGKV1-5 \| up \| immune system process(GO:0002376);metabolic process(GO:0008152);cellular process(GO:0009987);response to stimulus(GO:0050896);localization(GO:0051179);biological regulation(GO:0065007) \| \| IGHM \| up \| immune system process(GO:0002376);cellular process(GO:0009987);biological process involved in interspecies interaction between organisms(GO:0044419);response to stimulus(GO:0050896);biological regulation(GO:0065007) \| \| IGHV3-72 \| up \| -- \| \| IGKV2-24 \| up \| immune system process(GO:0002376);metabolic process(GO:0008152);cellular process(GO:0009987);response to stimulus(GO:0050896);localization(GO:0051179);biological regulation(GO:0065007) \| \| HRG \| up \| immune system process(GO:0002376);cellular process(GO:0009987);biological process involved in interspecies interaction between organisms(GO:0044419);response to stimulus(GO:0050896);localization(GO:0051179);biological regulation(GO:0065007) \| \| IGHV3-7 \| up \| -- \| \| IGKV1-8 \| up \| immune system process(GO:0002376);metabolic process(GO:0008152);cellular process(GO:0009987);biological process involved in interspecies interaction between organisms(GO:0044419);response to stimulus(GO:0050896);localization(GO:0051179);biological regulation(GO:0065007) \| \| IGLV3-19 \| up \| -- \| \| IGHV3-15 \| up \| -- \| \| THBS4 \| up \| behavior(GO:0007610);cellular process(GO:0009987);biological adhesion(GO:0022610);developmental process(GO:0032502);locomotion(GO:0040011);response to stimulus(GO:0050896);biological regulation(GO:0065007) \| \| IGLV1-47 \| up \| immune system process(GO:0002376);cellular process(GO:0009987);biological process involved in interspecies interaction between organisms(GO:0044419);response to stimulus(GO:0050896);biological regulation(GO:0065007) \| \| VCAN \| up \| metabolic process(GO:0008152);cellular process(GO:0009987);biological adhesion(GO:0022610);developmental process(GO:0032502);locomotion(GO:0040011);biological regulation(GO:0065007) \| \| LGALS3BP \| up \| metabolic process(GO:0008152);cellular process(GO:0009987);biological adhesion(GO:0022610);response to stimulus(GO:0050896);localization(GO:0051179);biological regulation(GO:0065007) \| \| IGKV1-16 \| up \| immune system process(GO:0002376);metabolic process(GO:0008152);cellular process(GO:0009987);biological process involved in interspecies interaction between organisms(GO:0044419);response to stimulus(GO:0050896);localization(GO:0051179);biological regulation(GO:0065007) \| \| IGHV3-9 \| up \| immune system process(GO:0002376);metabolic process(GO:0008152);cellular process(GO:0009987);multicellular organismal process(GO:0032501);biological process involved in interspecies interaction between organisms(GO:0044419);response to stimulus(GO:0050896);localization(GO:0051179);biological regulation(GO:0065007) \| \| IGLON \| up \| -- \| \| FCN2 \| up \| immune system process(GO:0002376);metabolic process(GO:0008152);cellular process(GO:0009987);biological process involved in interspecies interaction between organisms(GO:0044419);response to stimulus(GO:0050896);localization(GO:0051179);biological regulation(GO:0065007) \| \| IGHV1-18 \| up \| -- \| \| IGLV3-25 \| up \| -- \| \| F13A1 \| up \| metabolic process(GO:0008152);cellular process(GO:0009987);response to stimulus(GO:0050896);localization(GO:0051179) \| \| IGHV1-69D \| up \| -- \| \| JCHAIN \| up \| immune system process(GO:0002376);cellular process(GO:0009987);multicellular organismal process(GO:0032501);biological process involved in interspecies interaction between organisms(GO:0044419);response to stimulus(GO:0050896);localization(GO:0051179);biological regulation(GO:0065007) \| |  |  |
| --- | --- | --- | --- | --- | --- | --- | --- | --- | --- | --- | --- | --- | --- | --- | --- | --- | --- | --- | --- | --- | --- | --- | --- | --- | --- | --- | --- | --- | --- | --- | --- | --- | --- | --- | --- | --- | --- | --- | --- | --- | --- | --- | --- | --- | --- | --- | --- | --- | --- | --- | --- | --- | --- | --- | --- | --- | --- | --- | --- | --- | --- | --- | --- | --- | --- | --- | --- | --- | --- | --- | --- | --- | --- | --- | --- | --- | --- | --- | --- | --- | --- | --- | --- | --- | --- | --- | --- | --- | --- | --- | --- | --- | --- | --- | --- | --- | --- | --- | --- | --- | --- | --- | --- | --- | --- | --- | --- | --- | --- | --- | --- | --- | --- | --- | --- | --- | --- | --- | --- | --- | --- | --- | --- | --- | --- | --- | --- | --- | --- | --- | --- | --- | --- | --- | --- | --- | --- | --- | --- | --- | --- | --- | --- | --- | --- | --- | --- | --- | --- | --- | --- | --- | --- | --- | --- | --- | --- | --- | --- | --- | --- | --- | --- | --- | --- | --- | --- | --- | --- | --- | --- | --- | --- | --- | --- | --- | --- | --- | --- | --- | --- | --- | --- | --- | --- | --- | --- | --- | --- | --- | --- | --- | --- | --- | --- | --- | --- | --- | --- | --- | --- | --- | --- | --- | --- | --- | --- | --- | --- | --- | --- | --- |

**Table S4: KEGG enrichment of differentially expressed proteins.**

| **Pathway ID** | **Pathway Name** | **P-value** | **Protein Number** |
| --- | --- | --- | --- |
| ko05171 | Coronavirus disease - COVID-19 | 0.067649 | 21 |
| ko05322 | Systemic lupus erythematosus | 0.028726 | 20 |
| ko04020 | Calcium signaling pathway | 0.004321 | 20 |
| ko04145 | Phagosome | 0.006638 | 20 |
| ko05414 | Dilated cardiomyopathy | 0.005004 | 19 |
| ko05340 | Primary immunodeficiency | 0.009478 | 19 |
| ko04672 | Intestinal immune network for IgA production | 0.009478 | 19 |
| ko04151 | PI3K-Akt signaling pathway | 0.028386 | 19 |
| ko05416 | Viral myocarditis | 0.007154 | 18 |
| ko04613 | Neutrophil extracellular trap formation | 0.032767 | 18 |
| ko05135 | Yersinia infection | 0.007154 | 18 |
| ko05152 | Tuberculosis | 0.010917 | 18 |
| ko05150 | Staphylococcus aureus infection | 0.176634 | 18 |
| ko05146 | Amoebiasis | 0.03849 | 18 |
| ko05169 | Epstein-Barr virus infection | 0.008871 | 18 |
| ko04072 | Phospholipase D signaling pathway | 0.007154 | 18 |
| ko04064 | NF-kappa B signaling pathway | 0.007154 | 18 |
| ko05202 | Transcriptional misregulation in cancer | 0.018514 | 17 |
| ko05330 | Allograft rejection | 0.012451 | 17 |
| ko05310 | Asthma | 0.012451 | 17 |
| ko05323 | Rheumatoid arthritis | 0.012451 | 17 |
| ko05320 | Autoimmune thyroid disease | 0.012451 | 17 |
| ko04666 | Fc gamma R-mediated phagocytosis | 0.012451 | 17 |
| ko04664 | Fc epsilon RI signaling pathway | 0.012451 | 17 |
| ko04650 | Natural killer cell mediated cytotoxicity | 0.012451 | 17 |
| ko04662 | B cell receptor signaling pathway | 0.015241 | 17 |
| ko04640 | Hematopoietic cell lineage | 0.022326 | 17 |
| ko05140 | Leishmaniasis | 0.018514 | 17 |
| ko05143 | African trypanosomiasis | 0.059706 | 17 |
| ko05415 | Diabetic cardiomyopathy | 0.000826 | 10 |
| ko04933 | AGE-RAGE signaling pathway in diabetic complications | 0.012695 | 9 |
| ko04611 | Platelet activation | 0.023371 | 9 |
| ko04974 | Protein digestion and absorption | 0.040848 | 8 |
| ko04926 | Relaxin signaling pathway | 0.01662 | 8 |
| ko04610 | Complement and coagulation cascades | 0.943686 | 7 |
| ko01100 | Metabolic pathways | 0.781928 | 5 |
| ko05417 | Lipid and atherosclerosis | 0.126702 | 4 |
| ko04141 | Protein processing in endoplasmic reticulum | 0.206312 | 4 |
| ko05020 | Prion disease | 0.440057 | 4 |
| ko04514 | Cell adhesion molecules | 0.126702 | 4 |
| ko04979 | Cholesterol metabolism | 0.502399 | 3 |
| ko04918 | Thyroid hormone synthesis | 0.045944 | 3 |
| ko05132 | Salmonella infection | 0.170184 | 3 |
| ko05205 | Proteoglycans in cancer | 0.385569 | 2 |
| ko05200 | Pathways in cancer | 0.774001 | 2 |
| ko00010 | Glycolysis / Gluconeogenesis | 0.310243 | 2 |
| ko05418 | Fluid shear stress and atherosclerosis | 0.233484 | 2 |
| ko05410 | Hypertrophic cardiomyopathy | 0.233484 | 2 |
| ko04510 | Focal adhesion | 0.7352 | 2 |
| ko04934 | Cushing syndrome | 0.158468 | 2 |
| ko04936 | Alcoholic liver disease | 0.641339 | 2 |
| ko04925 | Aldosterone synthesis and secretion | 0.089804 | 2 |
| ko04927 | Cortisol synthesis and secretion | 0.158468 | 2 |
| ko03320 | PPAR signaling pathway | 0.457364 | 2 |
| ko01230 | Biosynthesis of amino acids | 0.158468 | 2 |
| ko01200 | Carbon metabolism | 0.233484 | 2 |
| ko04612 | Antigen processing and presentation | 0.310243 | 2 |
| ko05168 | Herpes simplex virus 1 infection | 0.385569 | 2 |
| ko05012 | Parkinson disease | 0.457364 | 2 |
| ko05022 | Pathways of neurodegeneration - multiple diseases | 0.641339 | 2 |
| ko05014 | Amyotrophic lateral sclerosis | 0.641339 | 2 |
| ko04066 | HIF-1 signaling pathway | 0.585752 | 2 |
| ko00350 | Tyrosine metabolism | 0.338302 | 1 |
| ko05207 | Chemical carcinogenesis - receptor activation | 0.646519 | 1 |
| ko05215 | Prostate cancer | 0.338302 | 1 |
| ko05225 | Hepatocellular carcinoma | 0.462551 | 1 |
| ko05412 | Arrhythmogenic right ventricular cardiomyopathy | 0.462551 | 1 |
| ko04210 | Apoptosis | 0.462551 | 1 |
| ko04218 | Cellular senescence | 0.462551 | 1 |
| ko04810 | Regulation of actin cytoskeleton | 0.768475 | 1 |
| ko04520 | Adherens junction | 0.186147 | 1 |
| ko04530 | Tight junction | 0.563912 | 1 |
| ko04261 | Adrenergic signaling in cardiomyocytes | 0.186147 | 1 |
| ko04270 | Vascular smooth muscle contraction | 0.462551 | 1 |
| ko04970 | Salivary secretion | 0.186147 | 1 |
| ko04971 | Gastric acid secretion | 0.338302 | 1 |
| ko04975 | Fat digestion and absorption | 0.563912 | 1 |
| ko04977 | Vitamin digestion and absorption | 0.646519 | 1 |
| ko04931 | Insulin resistance | 0.186147 | 1 |
| ko04921 | Oxytocin signaling pathway | 0.186147 | 1 |
| ko04924 | Renin secretion | 0.186147 | 1 |
| ko04614 | Renin-angiotensin system | 0.186147 | 1 |
| ko04919 | Thyroid hormone signaling pathway | 0.186147 | 1 |
| ko04915 | Estrogen signaling pathway | 0.462551 | 1 |
| ko04913 | Ovarian steroidogenesis | 0.563912 | 1 |
| ko04714 | Thermogenesis | 0.186147 | 1 |
| ko04120 | Ubiquitin mediated proteolysis | 0.186147 | 1 |
| ko03060 | Protein export | 0.186147 | 1 |
| ko03018 | RNA degradation | 0.338302 | 1 |
| ko00510 | N-Glycan biosynthesis | 0.462551 | 1 |
| ko04670 | Leukocyte transendothelial migration | 0.186147 | 1 |
| ko04620 | Toll-like receptor signaling pathway | 0.338302 | 1 |
| ko04657 | IL-17 signaling pathway | 0.338302 | 1 |
| ko05100 | Bacterial invasion of epithelial cells | 0.338302 | 1 |
| ko05131 | Shigellosis | 0.462551 | 1 |
| ko05130 | Pathogenic Escherichia coli infection | 0.81292 | 1 |
| ko05133 | Pertussis | 0.949099 | 1 |
| ko05144 | Malaria | 0.563912 | 1 |
| ko05142 | Chagas disease | 0.768475 | 1 |
| ko05167 | Kaposi sarcoma-associated herpesvirus infection | 0.338302 | 1 |
| ko05164 | Influenza A | 0.338302 | 1 |
| ko05170 | Human immunodeficiency virus 1 infection | 0.462551 | 1 |
| ko05163 | Human cytomegalovirus infection | 0.462551 | 1 |
| ko05165 | Human papillomavirus infection | 0.901933 | 1 |
| ko00140 | Steroid hormone biosynthesis | 0.563912 | 1 |
| ko05010 | Alzheimer disease | 0.848994 | 1 |
| ko04015 | Rap1 signaling pathway | 0.186147 | 1 |
| ko04310 | Wnt signaling pathway | 0.186147 | 1 |
| ko04390 | Hippo signaling pathway | 0.462551 | 1 |
| ko04080 | Neuroactive ligand-receptor interaction | 0.713774 | 1 |
| ko04512 | ECM-receptor interaction | 0.936592 | 1 |
| ko04137 | Mitophagy - animal | 0.186147 | 1 |
| ko04144 | Endocytosis | 0.713774 | 1 |
